# Dual RNA 3’-end processing of H2A.X messenger RNA maintains DNA damage repair throughout the cell cycle

**DOI:** 10.1038/s41467-020-20520-6

**Published:** 2021-01-13

**Authors:** Esther Griesbach, Margarita Schlackow, William F. Marzluff, Nick J. Proudfoot

**Affiliations:** 1 Sir William Dunn School of Pathology, University of Oxford, South Parks Road, Oxford, OX1 3RE UK; 2 Department of Biochemistry and Biophysics, University of North Carolina, Chapel Hill, NC 27599 USA

**Keywords:** RNA, Gene regulation, DNA damage and repair, RNA metabolism

## Abstract

Phosphorylated H2A.X is a critical chromatin marker of DNA damage repair (DDR) in higher eukaryotes. However, H2A.X gene expression remains relatively uncharacterised. Replication-dependent (RD) histone genes generate poly(A)- mRNA encoding new histones to package DNA during replication. In contrast, replication-independent (RI) histone genes synthesise poly(A)+ mRNA throughout the cell cycle, translated into histone variants that confer specific epigenetic patterns on chromatin. Remarkably *H2AFX*, encoding H2A.X, is a hybrid histone gene, generating both poly(A)+ and poly(A)- mRNA isoforms. Here we report that the selective removal of either mRNA isoform reveals different effects in different cell types. In some cells, RD H2A.X poly(A)- mRNA generates sufficient histone for deposition onto DDR associated chromatin. In contrast, cells making predominantly poly(A)+ mRNA require this isoform for de novo H2A.X synthesis, required for efficient DDR. This highlights the importance of differential H2A.X mRNA 3’-end processing in the maintenance of effective DDR.

## Abstract (web summary)

H2A.X histone variant gene encodes poly(A)+ and poly(A)- mRNA isoforms which are differentially expressed depending on cell lines. Here the authors show that upon DNA damage, cells expressing more poly(A)+ isoform require this isoform for de novo H2A.X synthesis while cells with more poly(A)- isoform have sufficient H2A.X present in chromatin.

## Introduction

Metazoans have evolved specific spatial and structural mechanisms to fine-tune the expression of core histone genes with DNA replication. Notably all five canonical histone proteins (H2A, H2B, H3, H4 and the linker histone H1) are expressed from multi-copy gene clusters^1^ of replication-dependent (RD) histone genes to ensure their large scale, stoichiometric production in the S-phase of the cell cycle. The molecular basis for this coordinated regulation lies in the assembly of histone locus bodies (HLB)^2,3^ over RD histone genes that act to concentrate specific transcription and pre-mRNA processing factors at their sites of action. One such critical HLB component is the histone gene-specific transcription factor NPAT, activated by phosphorylation in late G1 by CyclinE/Cdk2^4–6^. In mammalian cells, transcriptional activation and 3’-end processing combines to cause a 30-fold induction of histone mRNA production during the S-phase^7,8^. As these RD histone genes do not have introns and lack a poly(A) tail, only a single endonucleolytic cleavage reaction is required to generate their mRNA. This RNA 3’-end processing reaction requires two *cis*-elements, a conserved stem-loop (SL) structure followed by a purine-rich region called the histone downstream element (HDE). The SL is bound by the S-phase-specific stem-loop binding protein (SLBP), which is required both for RD histone pre-mRNA 3’-end processing and subsequent mRNA translation and stability^9,10^. The HDE is recognised by base pairing to U7 snRNA as part of a small nuclear ribonucleoprotein (U7 snRNP)^11^. Further 3’-end processing factors are then recruited, including CPSF-73, which catalyses the endonucleolytic cleavage reaction^12^. The full structure of this assembled 3’-end processing complex has been recently established by cryoEM^13^, confirming a remarkable cooperation between histone-specific and a subset of cleavage and polyadenylation (CPA) factors, especially CPSF-73. Once DNA replication is concluded at the end of the S-phase, RD histone mRNA is rapidly degraded by a mechanism involving active translation and SLBP^9^.

Some atypical histone proteins are also required outside of the S-phase, provided by replication-independent (RI) histone genes such as H2A.Z. These encode variant histones that confer a range of epigenetic regulatory processes^14^. RI histone genes are not linked to each other and are distinct from RD histone genes with their pre-mRNA processed by CPA factors like bulk poly(A) mRNA. Under certain circumstances, RD histone genes can also express low levels of polyadenylated mRNA. This often occurs when U7-dependent processing is deficient, leading to RNA polymerase II (Pol II) read-through and subsequent 3’-end processing at a downstream cryptic polyadenylation site (PAS)^15–19^. In addition, it has been shown that some RD histone genes express extended polyadenylated mRNA even under normal physiological conditions, such as upon terminal differentiation^20^ or following DNA damage^21^.

The *H2AFX* gene, encoding the H2A variant H2A.X is unique among histone genes in that it employs an intermediate pattern of mRNA 3’-end formation. It expresses both S-phase-specific mRNA ending in the typical SL structure of RD histone mRNA (H2A.X SL mRNA) and RI mRNA ending in a poly(A) tail dictated by a PAS (H2A.X poly(A) mRNA)^22^. Interestingly, *H2AFX* is not located in the histone gene clusters (i.e. outside of HLBs) and has a different promoter structure compared to RD histone genes^23^ that enables *H2AFX* transcription also outside S-phase^24^. The H2A.X protein is well-known for its function in the DNA damage response. In mammals it comprises 2–25% of the total chromatin H2A pool^25,26^. Its protein structure is very similar to core H2A apart from a C-terminal extension containing a conserved SQ[E/D]Φ motif. DNA damage leads to the phosphorylation of the serine residue in this motif (Ser139) by PI3K-like kinases including ATM, ATR and DNA-PK, forming γH2A.X^27–29^. γH2A.X specifically forms on nucleosomes at the damage site and then spreads to wider regions up to megabase lengths of DNA^30,31^. It has been suggested that these γH2A.X chromatin patches act as a platform for the binding of DNA repair factors^32,33^. Even though γH2A.X is dispensable for the initial recognition of DSBs, it is required to maintain the stable association of repair factors at DSB sites^34,35^. This is thought to be critical to tether broken DNA ends together^36^.

We sought to better understand the regulation and molecular consequences of alternative 3’-end formation of *H2AFX* transcripts. While previous work has focused on H2A.X protein, the differential function of its two H2A.X mRNA isoforms in the DNA damage response has not been characterised. Since it has been shown that different human cell lines express different relative ratios of H2A.X mRNA isoforms^24^, we specifically depleted each mRNA isoform in these different cell lines by use of RNA interference (RNAi) or CRISPR-Cas9 gene editing. Our results reveal that when cells express higher levels of H2A.X poly(A) mRNA, such as HeLa and RPE-1, they rely on the de novo incorporation of H2A.X into chromatin from this mRNA throughout the whole cell cycle to efficiently signal response to DNA damage by γH2A.X. In contrast, cells that express high levels of the S-phase-specific H2A.X SL mRNA, such as HCT-116 and Jurkat cells, incorporate higher levels of H2A.X into the chromatin during DNA replication. This is sufficient for efficient γH2A.X signalling following DNA damage induction throughout the rest of the cell cycle. It is apparent that the flexible regulation of these two H2A.X mRNA isoforms described in this study is critical to maintain efficient DNA repair processes in different cell types.

## Results

### *H2AFX* expresses both poly(A) and SL mRNA isoforms

**Fig. 1:**
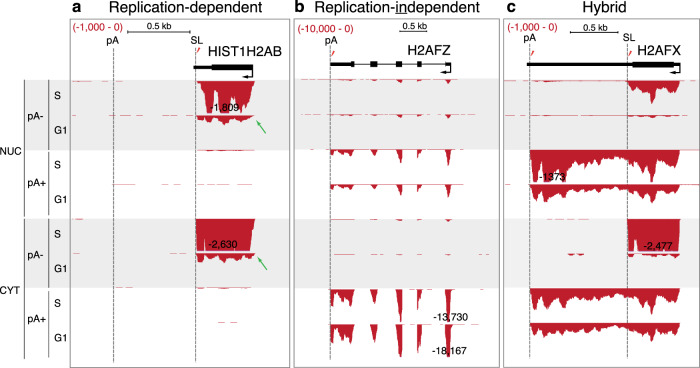
H2AFX expresses poly(A)**+** and poly(A)- mRNA. HeLa cell RNA-seq reads aligned to human genome. Scale indicated in red in upper left corner. pA-: rRNA-depleted poly(A)- RNA. pA+ : poly(A)+ RNA. S and G1: S- and G1-phase-enriched cells. SL: U7-dependent cleavage site. pA: polyadenylation site. Black arrow: direction of Pol II transcription. Maximum read number indicated. Green arrows: poly(A)- histone mRNA in G1 fractions derived from 3% S-phase cell contamination (Supplementary Fig. 1b). **a** RNA-seq profile of a typical replication-dependent histone gene. **b** Example of a replication-independent histone gene. **c** H2AFX gene with replication-dependent and independent features.

**Fig. 2:**
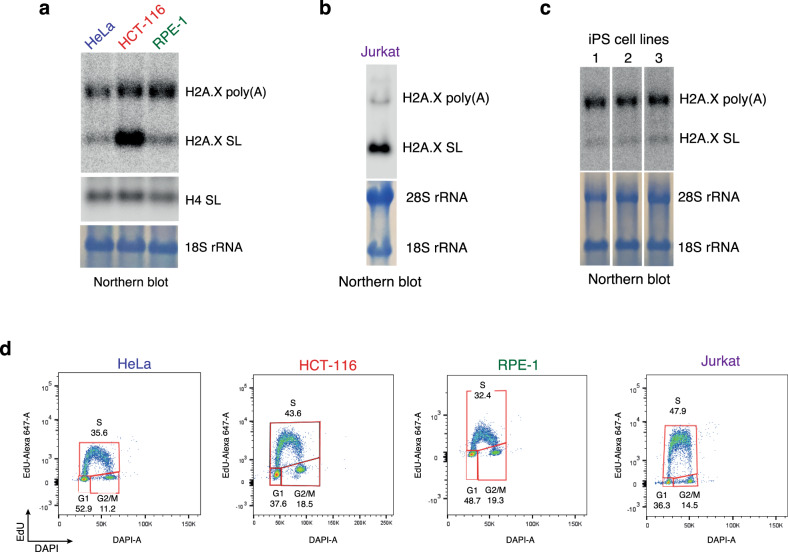
*H2AFX* expresses both H2A.X poly(A) and SL mRNA isoforms. **a** H2A.X mRNA northern blot of total RNA from asynchronous HeLa, HCT-116 and RPE-1 cells. H4 SL: core H4 histone SL mRNA. 18S rRNA used as loading control. **b** H2A.X mRNA northern blot of total RNA from asynchronous Jurkat cells. 28S and 18S rRNA used as loading controls. **c** H2A.X mRNA northern blot of total RNA from three different human iPS cell lines. 28S and 18S rRNA used as loading controls. **d** EdU labelling of asynchronous HeLa, HCT-116, RPE-1 and Jurkat cells showing DAPI fluorescent intensity on x-axis and Alexa 647-EdU fluorescent intensity on y-axis.

We initially catalogued the transcription of histone genes in S- and G1-phase HeLa cells by cell fractionation and RNA-seq analysis (Supplementary Fig. 1a–c). Our goal was to identify RD-histone genes that in addition to the typical SL mRNA also express a cytoplasmic poly(A) mRNA isoform. While most RD histone genes only express SL mRNA in the S-phase (Fig. 1a, Supplementary Fig. 1d and Supplementary Table 1) and RI histone genes generate poly(A) mRNA throughout the cell cycle (Fig. 1b), some RD histone genes express both SL and poly(A) mRNA (Fig. 1c, Supplementary Fig. 1e, f and Table 1). *H2AFX* is the only example of such a “hybrid” histone gene that expresses relatively high levels of poly(A) mRNA compared to S-phase-specific SL mRNA (Fig. 1c). This encodes the variant histone H2A.X associated with the DNA damage response (DDR). The existence of the two H2A.X mRNA isoforms has been previously documented^22^, even though their biological relevance in DDR remains unknown. It is also known that different cell lines express different amounts of H2A.X mRNA isoforms^24^. We therefore elected to study in detail HeLa and RPE-1 cells, that predominantly express H2A.X poly(A) mRNA and compare this to HCT-116 and Jurkat cells that express more H2A.X SL mRNA (Fig. 2a, b). RPE-1 cells are diploid and immortalised, derived from retinal pigment epithelium. Human induced pluripotent stem (iPS) cells, like HeLa cells, express mostly H2A.X poly(A) mRNA (Fig. 2c). To quantitatively distinguish the two H2A.X mRNA isoforms, we used northern blotting with a probe that detects both the SL and poly(A) H2A.X mRNA. Core H4 histone SL mRNA was used as a positive control and methylene blue staining of 18S ribosomal RNA (rRNA) as a loading control. It should be noted that, asynchronous HCT-116 and Jurkat cells have about 10% more S-phase cells compared to HeLa and RPE-1, based on EdU labelling and flow cytometry of each cell type (Fig. 2d, flow cytometry gating strategy shown in Supplementary Fig. 2a).

### Cell cycle profiles of H2A.X SL and poly(A) mRNA isoforms

**Fig. 3:**
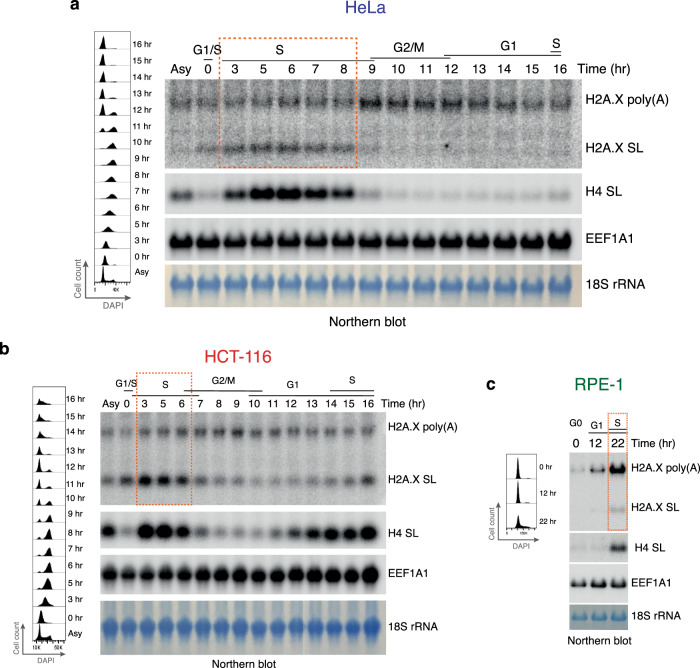
Comparative H2A.X mRNA cell cycle distributions. **a** Cell cycle synchronisation of HeLa cells by double thymidine block (DTB). Cell cycle profiles (time following DTB release) showing DAPI fluorescent intensity on x-axis and cell count on y-axis (left). H2A.X mRNA northern blot of total RNA (right). Asy: asynchronous cells. Cell cycle phases determined by flow cytometry are indicated. H4 SL: core H4 histone SL mRNA. EEF1A1 mRNA and 18S rRNA used as loading controls. Orange box: S-phase. **b** As in **a** but for HCT-116 cells. **c** Cell cycle profiles of RPE-1 cells synchronised by contact inhibition (time after block release indicated) showing DAPI fluorescent intensity on x-axis and cell count on y-axis (left). H2A.X mRNA northern blot of total RNA from synchronised RPE-1 cells (right). H4 SL: core H4 histone SL mRNA. EEF1A1 mRNA and 18S rRNA used as loading controls.

To characterise the differential expression patterns of H2A.X mRNA isoforms throughout the cell cycle, HeLa and HCT-116 cells were synchronised by double thymidine block (DTB) at the G1/S-phase border. Following block release, they were collected at the indicated time points and analysed for cell cycle stage (Fig. 3a, b). RPE-1 cells were instead synchronised in G0 by contact inhibition, as described previously^37^, and time points were subsequently collected at 12 hr (G1) and 22 hr (~50% S-phase based on EdU labelling) after cell cycle re-entry (Fig. 3c). Total RNA was extracted from each cell cycle time point and used for northern blotting. Eukaryotic Translation Elongation Factor 1 Alpha 1 (EEF1A1) mRNA was used as an additional loading control. Both HeLa and HCT-116 cells express H2A.X poly(A) mRNA throughout the cell cycle, with an increase in G2/M (Fig. 3a, b), as reported previously^38,39^. As the DTB-synchronised cells progressed through S-phase, the amount of H2A.X poly(A) mRNA increased (see change from 3 hr to 6 hr time points in Fig. 3a, b), suggesting that a fraction of H2A.X poly(A) mRNA is synthesised during the S-phase. Similarly, in RPE-1 cells, H2A.X poly(A) mRNA was expressed at very low levels in G0 and increased as cells progressed to G1 and into S-phase (Fig. 3c). In contrast, H2A.X SL mRNA paralleled S-phase-specific H4 SL mRNA expression in all three cell lines increasing at the onset of S-phase and rapidly decreasing with S-phase completion. The half-life of the H2A.X SL mRNA was previously found to be comparable to bulk histone mRNA (35–40 min)^24,40^, and the H2A.X poly(A) a 6-fold longer half-life of about 4 hr^41^. Notably, S-phase HCT-116 cells expressed higher H2A.X SL mRNA levels as compared to HeLa and RPE-1 cells. This is consistent with increased H2A.X SL mRNA synthesis during the S-phase in HCT-116 cells (compare H2A.X poly(A) versus SL mRNA in orange boxes in Fig. 3a–c). Furthermore, this increase was specific for H2A.X SL mRNA as H4 SL mRNA levels were comparable between the three cell lines (Fig. 2a). This excludes the possibility that increased H2A.X SL mRNA levels as observed in asynchronous cell populations are simply due to differential cell cycle phase distributions.

*H2AFX* is located outside of the histone gene clusters and its promoter is not bound by NPAT at significant levels^42^ (Supplementary Fig. 2b). Thus, it is likely that H2A.X mRNA expression (including H2A.X SL mRNA) is regulated independently of RD histone genes. Interestingly, we observed 1.5-fold higher U7 snRNA levels in HCT-116 than HeLa or RPE-1 cells (Supplementary Fig. 3a). Even though U7 snRNP is concentrated in the HLB, cells with increased U7 snRNA levels may potentially have more U7 snRNP in the nucleoplasm and so allow for more efficient processing at the H2A.X SL-HDE site. This may be consistent with higher levels of H2A.X SL mRNA in HCT-116 cells. To test this hypothesis, we overexpressed the U7 snRNP in HeLa cells using a novel strategy developed by Sarah Tisdale and Livio Pellizzoni (personal communication). They found that coexpression of the U7 snRNP-specific proteins LSM10 and LSM11 increases U7 snRNP biogenesis and function. We generated a HeLa Flp-In T-REx cell line that overexpresses LSM10 and LSM11 in a doxycycline-inducible manner. Notably, U7 snRNA levels increased 2-fold as a result of LSM10/11 overexpression (Supplementary Fig. 3b). Even so, we did not observe an increase in H2A.X SL mRNA levels (Supplementary Fig. 3c). These overexpression results imply that solely raising U7 snRNA levels is insufficient to increase H2A.X SL mRNA expression. Likely other factors will also contribute to the variable pattern of H2A.X mRNA alternative isoforms as seen in the different cell lines.

### SL and poly(A) mRNAs differ in H2A.X chromatin incorporation and DDR

**Fig. 4:**
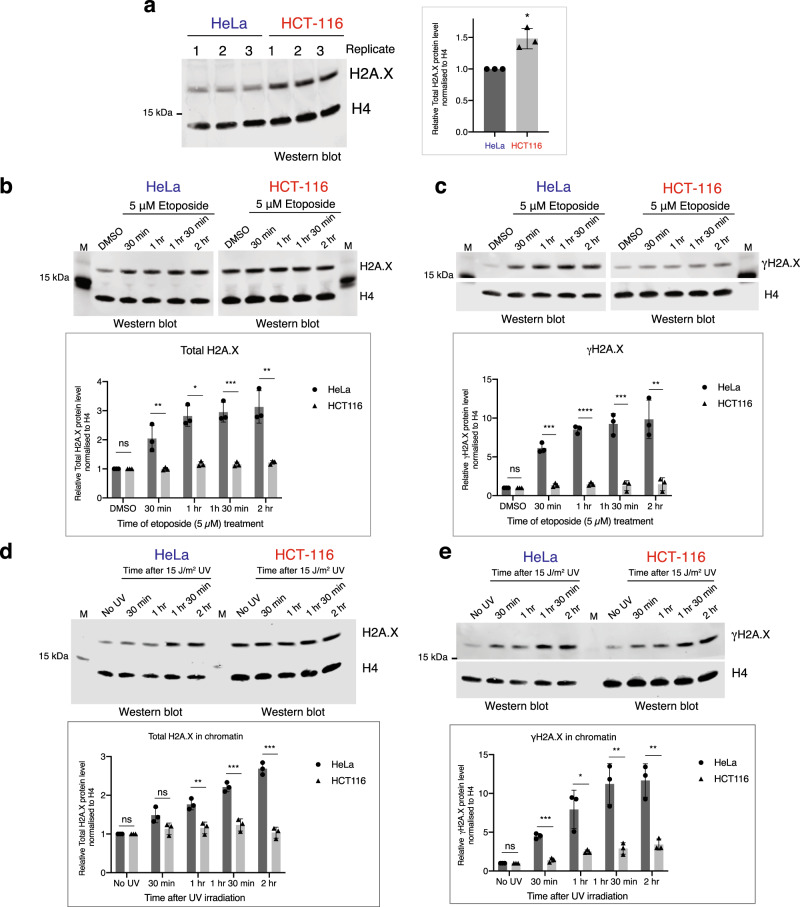
Analysis of H2A.X deposition in chromatin upon DNA damage induction. **a** H2A.X levels in chromatin. Western blot of fractionated chromatin from WT HeLa and HCT-116 cells. Total H2A.X and core H4 histone were detected with specific antibodies. Densitometry of western blot (right panel). Total H2A.X protein levels in HCT-116 cells are represented relative to HeLa cells and normalised to H4 histone. Mean ± SD presented for *n* = 3 independent experiments. Paired Student’s *t*-test, *P-value ≤ 0.05. **b** Western blot analysis of H2A.X in isolated chromatin from HeLa and HCT-116 cells treated with etoposide as indicated. H4 was used as loading control. M: protein size marker. Mean ± SD presented for *n* = 3 independent experiments. Unpaired Student’s *t*-test. ns: not significant *P*-value >0.05, *P-value ≤ 0.05, **P-value ≤ 0.01, ***P-value ≤ 0.001, ****P-value ≤ 0.0001. **c** As in **b** but for γH2A.X. **d** Western blot analysis of H2A.X in isolated chromatin from HeLa and HCT-116 cells after 15 J/m^2^ UV irradiation. H4 was used as loading control. M: protein size marker. Mean ± SD presented for *n* = 3 independent experiments. Unpaired Student’s *t*-test ns: not significant *P*-value >0.05, *P-value ≤ 0.05, **P-value ≤ 0.01, ***P-value ≤ 0.001. **e** As in **d** but for γH2A.X.

We wished to test whether the H2A.X SL and poly(A) mRNA isoforms play different roles in the deposition of H2A.X onto chromatin and whether this affected DDR. Chromatin was therefore purified from HeLa and HCT-116 cell nuclei (Supplementary Fig. 4a) and the relative amount of H2A.X in this fraction in different cells was compared to core H4 levels by western blotting. As shown in Fig. 4a, asynchronously growing HCT-116 cells have 1.5-fold more H2A.X in chromatin compared to HeLa cells. This indicates that the higher levels of H2A.X SL mRNA in HCT-116 cells allow for increased H2A.X deposition onto chromatin during DNA replication in S-phase.

It has been shown that DNA damage induction leads to de novo H2A.X deposition into chromatin^43^. We therefore tested if the higher basal levels of H2A.X seen in HCT-116 as compared to HeLa cell chromatin results in differences in H2A.X deposition following DNA damage. HeLa and HCT-116 cells were treated with the topoisomerase II inhibitor etoposide and levels of H2A.X in chromatin were assessed by western blot every 30 min for 2 hr. Notably, these increased in HeLa cells (up to 3-fold) but were unchanged in HCT-116 cells (Fig. 4b). This DNA damage effect was even more dramatic for γH2A.X with levels increasing up to 10-fold at later time points with HeLa but not with HCT-116 cells (Fig. 4c). The difference between these two cells lines in chromatin incorporation of H2A.X following DNA damage by etoposide treatment was mirrored by RPE-1 and Jurkat cells (Supplementary Fig. 4b–e). Thus, RPE-1 cells, which like HeLa cells, express higher levels of poly(A) than SL H2A.X mRNA showed a clear increase in H2A.X chromatin incorporation following etoposide treatment. In contrast, Jurkat cells showed only a small increase in H2A.X incorporation into chromatin following etoposide-induced DNA damage. However, Jurkat cells did show a significant increase in γH2A.X, reflecting modification of existing chromatin bound H2A.X protein in response to DNA damage. This is consistent with the high levels of H2A.X SL mRNA expressed in these cells. These experiments to measure H2A.X chromatin incorporation following DNA damage were reproducible between three biological repeats for all four cell types.

We also extended these data on DNA damage-induced H2A.X chromatin association to a different DNA damaging agent. Notably, irradiation of HeLa or HCT-116 cells with UV light displayed the same differential effects on H2A.X protein levels seen for etoposide treatment (Fig. 4d, e). Thus, HeLa cells showed de novo incorporation of H2A.X into chromatin while HCT-116 cells did not, but responded by modifying the chromatin bound H2A.X already present.

Taken together, these results show that cells expressing higher amounts of H2A.X SL mRNA such as HCT-116 and Jurkat, incorporate more H2A.X histone into the chromatin during DNA replication. These H2A.X levels are sufficient to signal DDR. In contrast, cells that express lower amounts of H2A.X SL mRNA, such as HeLa and RPE-1, incorporate less H2A.X into the chromatin during S-phase. Consequently, for efficient DDR, this lower basal H2A.X level must be compensated for by de novo H2A.X synthesis from H2A.X poly(A) mRNA and its incorporation into chromatin throughout the cell cycle.

### H2A.X poly(A) mRNA is required for cell cycle progression and genome stability

**Fig. 5:**
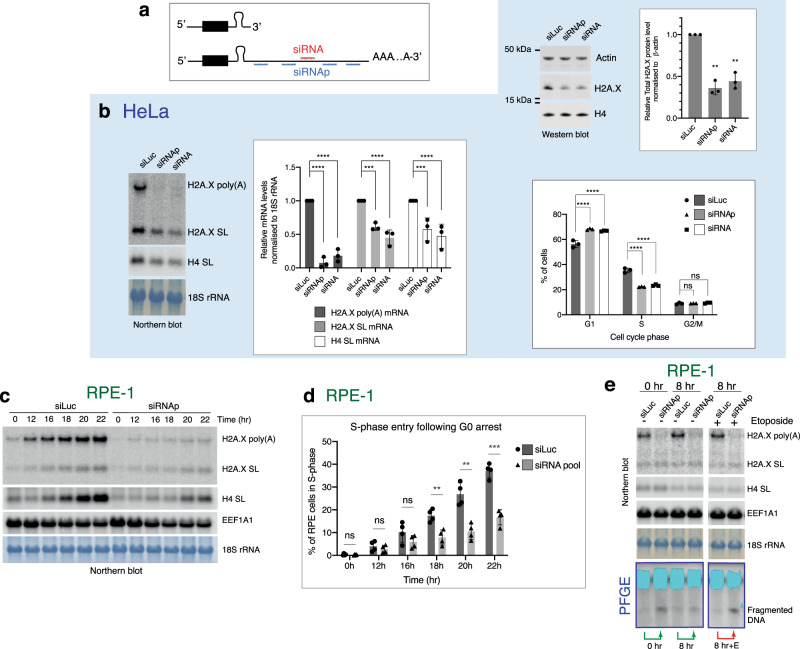
siRNA depletion of the H2A.X poly(A) mRNA. **a** Schematic of siRNA binding sites for 3’-UTR extension of H2A.X poly(A) mRNA. siRNA: single siRNA. siRNAp: pool of four different siRNAs. **b** H2A.X mRNA northern blot of total RNA and quantification from HeLa cells treated with indicated siRNAs for 72 hr. Mean ± SD presented for *n* = 3 independent experiments, two-way ANOVA with Tukey’s multiple comparisons test. ***P-value ≤ 0.001, ****P-value ≤ 0.0001. H4 SL: core H4 histone SL mRNA. 18S rRNA used as loading control (left). Total protein extract western blot and quantification. Actin and core histone H4 are controls. H2A.X indicates total H2A.X protein levels. The two 15 kDa marks indicate where the membrane was cut. Mean ± SD presented for *n* = 3 independent experiments, paired Student’s *t*-test, **P-value ≤ 0.01 (top right). Percentages of cells in G1, S and G2/M analysed by flow cytometry in siRNA-treated HeLa cells. Staining for flow cytometry was performed using propidium iodide. Mean ± SD presented for *n* = 3 independent experiments, two-way ANOVA with Dunnett’s multiple comparisons test, ns: not significant *P*-value >0.05, ****P-value ≤ 0.0001 (bottom right). **c** H2A.X mRNA northern blot of total RNA from RPE-1 cells depleted of H2A.X poly(A) mRNA and synchronised by contact inhibition (time following block release). H4 SL: core H4 histone SL mRNA. EEF1A1 mRNA and 18S rRNA used as loading controls. **d** Percentage of S-phase cells in RPE-1 cell population treated with either siLuc (dark grey bars) or siRNAp (light grey bars) and synchronised by contact inhibition. X-axis represents time points in hours after release from cell cycle arrest and y-axis percentage of S-phase cells determined by EdU labelling followed by flow cytometry (Supplementary Fig. 5). Mean ± SD presented for *n* = 4 independent experiments. Unpaired Student’s t-test ns: not significant *P*-value > 0.05, **P-value ≤ 0.01, ***P-value ≤ 0.001. **e** H2A.X mRNA northern blot of total RNA from RPE-1 cells treated with indicated siRNAs and arrested for 4 days and harvested at indicated time points after resuming cell cycle. 5 μM etoposide added at 6 hr (G1) for a 2 hr period. H4 SL: core H4 histone SL mRNA. EEF1A1 mRNA and 18S rRNA used as loading controls. Same samples were analysed by PFGE. Dark blue box represents ethidium bromide-stained PFGE agarose gel. Light blue regions correspond to saturated DNA signal in gel well.

To dissect the function of the H2A.X poly(A) mRNA, we carried out selective depletion of this mRNA isoform by use of either a single siRNA (siRNA) or a pool of four different siRNAs (siRNAp) targeting the extended 3’-UTR (Fig. 5a). In HeLa cells, H2A.X poly(A) mRNA depletion was achieved following 48–72 hr of siRNA treatment and led to a substantial decrease in H2A.X protein levels (Fig. 5b). Surprisingly, both H2A.X SL and H4 SL mRNA decreased upon H2A.X poly(A) mRNA depletion. As histone SL mRNA is S-phase-specific, this suggested there was a reduction in the number of S-phase cells when H2A.X poly(A) mRNA was depleted. Flow cytometry analysis confirmed that the percentage of S-phase cells decreased by one third in cells treated with siRNAs (Fig. 5b). The number of cells in G1 was increased, suggesting a failure or delay in S-phase entry.

Similar results to HeLa were obtained with RPE-1 cells. We depleted the H2A.X poly(A) mRNA (Fig. 5c), synchronised the cells in G0 by contact inhibition and assessed the number of cells entering S-phase after release from the block (Fig. 5d). While indeed only half as many of the cells were able to enter S-phase when the H2A.X poly(A) mRNA was depleted (Fig. 5d), the cells that entered S-phase did not show an apparent DNA replication defect, as assessed by EdU incorporation followed by flow cytometry (Supplementary Fig. 5). This result is consistent with failure to initiate S-phase. To test if accumulated DNA damage might be responsible for this cell cycle defect in RPE-1 cells, pulsed-field gel electrophoresis (PFGE) was performed. As shown in Fig. 5e, cells depleted of the H2A.X poly(A) mRNA showed increased levels of fragmented DNA compared to the siLuc control in both G0 (0 hr) and G1 (8 hr) (green arrows in Fig. 5e). This effect was increased when the cells were treated with etoposide (red arrow in Fig. 5e). Overall, the above data demonstrate that the H2A.X poly(A) mRNA is important for cell cycle progression and genome stability in cells expressing higher amounts of H2A.X poly(A) mRNA, such as HeLa and RPE-1.

### γH2A.X signalling is impaired in HeLa cells depleted of the H2A.X poly(A) mRNA

**Fig. 6:**
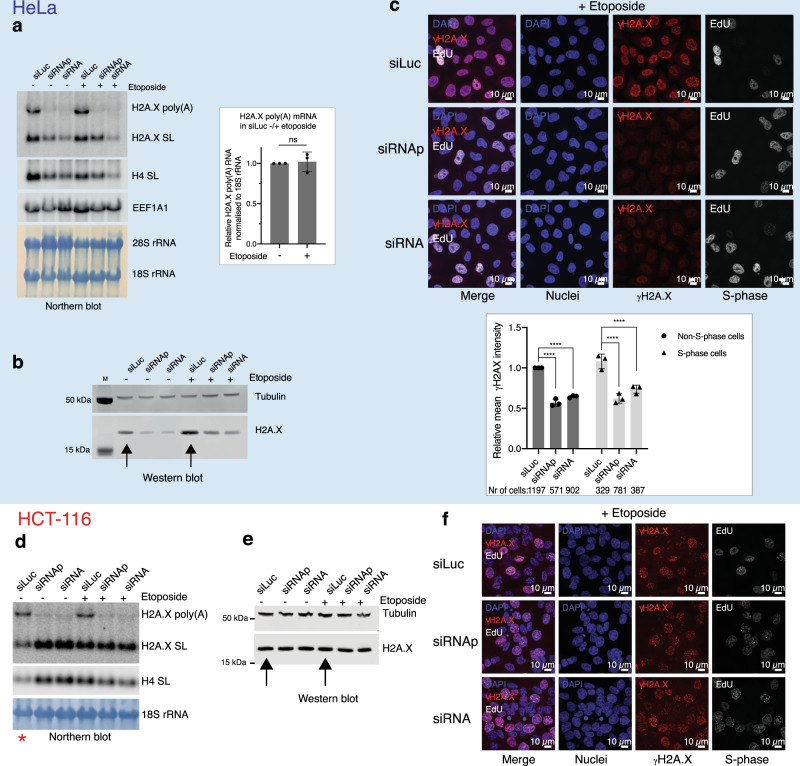
Effects of H2A.X poly(A) mRNA depletion under DNA damage conditions. **a** H2A.X mRNA northern blot of total RNA from HeLa cells treated with siRNA for 72 hr (left panel). H4 SL: core H4 histone SL mRNA. EEF1A1 mRNA, 28S and 18S rRNA used as loading controls. Etoposide treatment (5 μM) was performed for 2 hr before harvesting. Tubulin was used as loading control. H2A.X: total H2A.X protein. γH2A.X: H2A.X phosphorylated at Ser139. M: protein size marker. H2A.X poly(A) mRNA quantification in siLuc without and with etoposide by real-time PCR (right panel). Mean ± SD presented for *n* = 3 independent experiments, paired Student’s *t*-test, ns: not significant *P*-value >0.05. **b** Western blot of total protein extracts from HeLa cells as indicated. Etoposide treatment (5 μM) was performed for 2 hr before harvesting. Black arrows: total H2A.X protein in siLuc control cells before and after etoposide treatment. **c** IF of HeLa cells with etoposide treatment (5 μM for 2 hr before harvesting). Blue: DAPI (nuclei). Red: γH2A.X. White: EdU (S-phase cells). Quantification of nuclear γH2A.X signal in non-S-phase (black bars) and S-phase cells (grey bars) (bottom). Mean ± SD presented for *n* = 3 independent experiments, two-way ANOVA with Tukey’s multiple comparisons test. ****P-value ≤ 0.0001 (left). **d**–**f** As in **a**–**c**, respectively, but for HCT-116 cells. The red asterisk indicates unequal loading in lane 1 of **d**.

Since DNA damage leads to the phosphorylation of H2A.X, forming γH2A.X, we next tested whether depletion of H2A.X poly(A) mRNA affects γH2A.X signalling. HeLa cells depleted of H2A.X poly(A) mRNA were treated with etoposide for 2 hr before harvesting and H2A.X mRNA (Fig. 6a) as well as protein levels (Fig. 6b) were analysed. Notably, DNA damage induction led to an accumulation of total H2A.X protein (black arrows in Fig. 6b), which likely results from a change in H2A.X mRNA translation and/or protein stability as H2A.X poly(A) mRNA levels were unaffected by the DNA damage inducing agent (Fig. 6a). Similarly, S-phase cells depleted of the H2A.X poly(A) mRNA still express the same amount of H2A.X SL mRNA relative to H4 mRNA (Fig. 6a).

To distinguish between the γH2A.X signal in S-phase and non-S-phase HeLa cells we employed EdU cell imaging coupled with immunofluorescence (IF). IF analysis was performed on HeLa cells with or without siRNA mediated depletion of H2A.X poly(A) mRNA and stained with DAPI and an antibody against γH2A.X. Cells were further treated with etoposide as well as labelled with EdU to label S-phase cells (Fig. 6c). This revealed that γH2A.X nuclear foci are readily detected in an etoposide dependent manner. Notably, H2A.X poly(A) mRNA depletion led to a significant general decrease in γH2A.X IF signal in both non-S-phase and S-phase cells (Fig. 6c). This effect was rescued by stable expression of an siRNA-resistant wild-type (WT) *H2AFX* gene (siRNA binding site deleted) but not by a poly(A) site deletion mutant (Supplementary Fig. 6a–d). This latter experiment demonstrates that decreased γH2A.X signalling is specifically caused by lack of H2A.X poly(A) mRNA and not through indirect effects of perturbing H2A.X expression. We also confirmed that HeLa cells lacking the H2A.X poly(A) mRNA are deficient in H2A.X protein incorporation into chromatin upon DNA damage induction, supporting that it is de novo H2A.X synthesised from the H2A.X poly(A) mRNA that is incorporated into the damaged chromatin (Supplementary Fig. 6e).

In contrast to HeLa, in HCT-116 cells neither depletion of H2A.X poly(A) mRNA nor induction of DNA damage with etoposide affected total H2A.X protein levels (Fig. 6d, e; see black arrows). Similarly, IF analysis of γH2A.X in HCT-116 cells revealed no significant change in signal following H2A.X poly(A) mRNA depletion (Fig. 6f). Taken together, we demonstrate that HeLa cells expressing low levels of H2A.X SL mRNA rely on the poly(A) mRNA to provide sufficient H2A.X for incorporation into chromatin and efficient γH2A.X signalling. In contrast, continuously cycling cells that express higher levels of the H2A.X SL mRNA, such as HCT-116, are unaffected by the loss of the H2A.X poly(A) mRNA.

### Effect of selective H2A.X SL mRNA loss in HeLa and HCT-116 cells

**Fig. 7:**
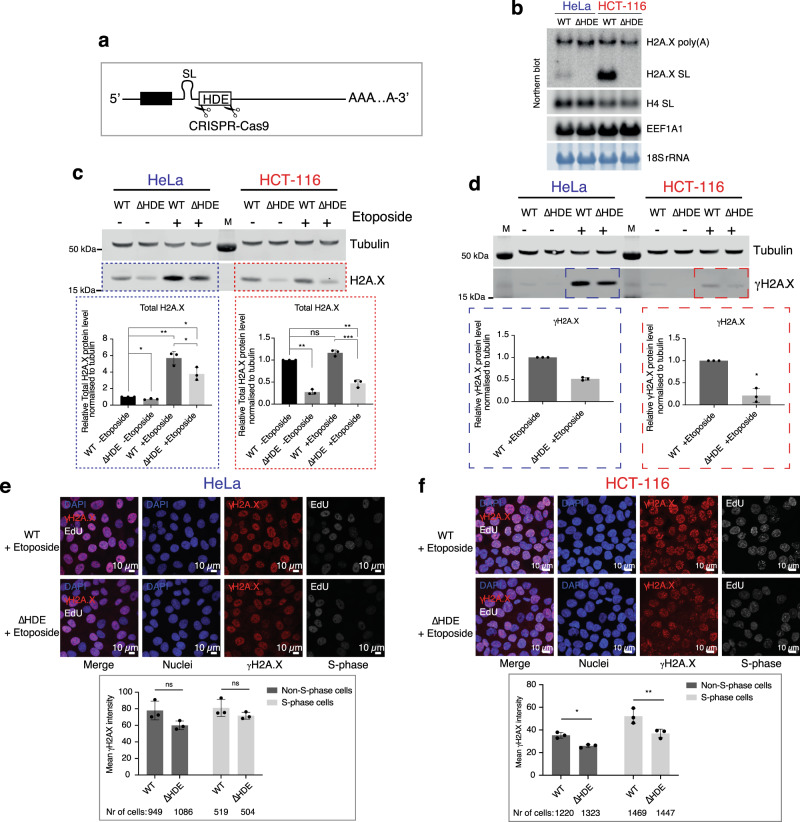
Effect of H2A.X SL mRNA loss in control versus DNA damage conditions. **a** Schematic of CRISPR HDE deletion. Two guide RNAs flanking HDE were used together with a ssDNA oligonucleotide template for homologous recombination. **b** H2A.X mRNA northern blot of total RNA from asynchronous WT (WT) and ∆HDE HeLa and HCT-116 cells. H4 SL: core H4 histone SL mRNA. EEF1A1 mRNA and 18S rRNA used as loading controls. **c**, **d** Western blots showing total H2A.X (**c**) and γH2A.X (**d**) protein levels in WT and ∆HDE HeLa and HCT-116 cells, with or without etoposide treatment. Tubulin: loading control. M: protein size marker. Blue (HeLa) and red (HCT-116) dotted lines highlight bands used for quantification, shown in graphs below. Quantifications represent total or γH2A.X levels normalised to tubulin and relative to the WT. Mean ± SD presented for *n* = 3 independent experiments. Paired Student’s *t*-test, ns: not significant *P*-value >0.05, *P-value ≤ 0.05, **P-value ≤ 0.01, ***P-value ≤ 0.001. **e** IF images of WT and ∆HDE HeLa cells treated with etoposide, labelled with EdU (white). Quantification of nuclear γH2A.X signal in etoposide-treated HeLa cells, comparing the γH2A.X intensity between WT and ∆HDE cells in non-S-phase or S-phase cells. 10 images of each sample were analysed for a total of three independent experiments. Mean ± SD presented for *n* = 3 independent experiments. Two-way ANOVA with Sidak’s multiple comparisons test. ns: not significant *P*-value >0.05, *P-value ≤ 0.05, **P-value ≤ 0.01. **f** Same as in **e** but for HCT-116 cells.

We next performed parallel experiments to study the function of H2A.X SL mRNA by its selective removal. Since the sequence of the H2A.X SL mRNA is shared with its extended polyadenylated counterpart, H2A.X SL mRNA does not contain any unique sequences that can be specifically targeted by RNAi. However, the 3’-end processing of H2A.X SL mRNA can be prevented by deletion of its HDE which is known to abolish U7-dependent processing of histone mRNA in mammalian cells^44^. Therefore, we targeted the *H2AFX* HDE for deletion by CRISPR-Cas9 technology in both HeLa and HCT-116 cells (Fig. 7a). A homozygous clone was obtained for each cell line (referred to as HeLa ΔHDE and HCT-116 ΔHDE). The homozygous HeLa ΔHDE clone also contained a 90 bp deletion downstream of the HDE deletion (Supplementary Fig. 7a). This deleted region does not contain any known sequence elements involved in the regulation of the H2A.X poly(A) mRNA and so was employed in further analysis. The SL structure is intact in both HeLa and HCT-116 ΔHDE clones so that the H2A.X poly(A) mRNA expressed in these cell lines still contains the SL, like the normal H2A.X poly(A) mRNA, which can still bind SLBP, and hence potentially stimulate translation^10^.

The HDE deletion completely abolished processing at the *H2AFX* U7-dependent processing site in both cell types (Fig. 7b). Consequently, the HDE deletion has a more severe effect on total levels of H2A.X mRNA in HCT-116 cells as H2A.X SL mRNA is the dominant isoform in this cell type. Interestingly, H2A.X poly(A) mRNA levels did not significantly increase in ΔHDE cells compared to WT cells (Fig. 7b and Supplementary Fig. 7b). This was surprising as we anticipated that HDE deletion would increase the level of read-through transcription up to the PAS, particularly in the HCT-116 cells, which produce large amounts of H2A.X SL mRNA. This argues against a simple competitive effect between these two adjacent mRNA 3’-end processing machineries. Furthermore, similarly to depletion of the H2A.X poly(A) mRNA (Fig. 5b-d), HDE deletion showed an effect on cell cycle distribution, as both cell lines had 10–25% fewer S-phase cells compared to the WT (Supplementary Fig. 7c).

H2A.X SL mRNA knock out resulted in decreased levels of total H2A.X protein in both cell types with, as expected, a stronger effect in HCT-116 (Fig. 7c). As observed previously (Fig. 6b), etoposide treatment led to an increase in total H2A.X protein levels in HeLa WT cells (about 5-fold) but not in HCT-116 cells (Fig. 7c). γH2A.X levels were also reduced in both ΔHDE cell lines as compared to the WT cells (Fig. 7d). As with total H2A.X, the effect was also more pronounced in HCT-116 cells as these mainly rely on H2A.X SL mRNA to generate H2A.X. To test whether the loss of the H2A.X SL mRNA has an effect on the cells outside of the S-phase, we measured γH2A.X levels by IF following etoposide treatment in S-phase and non-S-phase cells (EdU labelling). Both WT and ΔHDE HeLa and HCT-116 cells were tested. Nuclei were stained with DAPI and specific antibodies against γH2A.X (Fig. 7e, f). Nuclear γH2A.X signal was quantified in non-S-phase and in S-phase cells. In HeLa cells there was no significant difference in γH2A.X levels between WT and ΔHDE cells, independent of the cell cycle phase (Fig. 7e). In contrast, HCT-116 ΔHDE cells showed decreased γH2A.X signal in both S-phase and non-S-phase cells (Fig. 7f), indicating that in these cells the H2A.X made from the SL mRNA during DNA replication is required for γH2A.X signalling in later stages of the cell cycle.

**Fig. 8:**
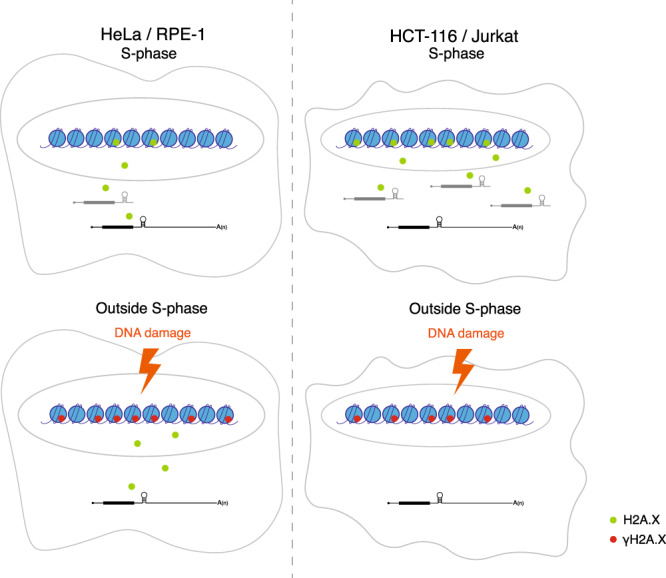
Model. In S-phase HCT-116 and Jurkat cells express increased H2A.X SL mRNA compared to HeLa and RPE-1 cells, so that they synthesise and incorporate more H2A.X protein into the newly made chromatin. Following DNA damage (etoposide treatment or UV irradiation), HeLa cells employ de novo H2A.X synthesis from H2A.X poly(A) mRNA to provide sufficient H2A.X for efficient γH2A.X signalling. In contrast, HCT-116 cells already contain sufficient H2A.X in chromatin to signal DNA damage, so obviating the need for de novo H2A.X synthesis.

Taken together, differences in severity of the ∆HDE mutation on HeLa and HCT-116 H2A.X expression correlate with the starting level of H2A.X SL mRNA. For HeLa cells that express only small levels of this mRNA isoform, little effect on H2A.X function was detectable. In contrast, HCT-116 cells that predominantly express the S-phase H2A.X SL mRNA were more severely affected, since they did not increase the expression of H2A.X poly(A) mRNA. Overall, our results establish the following mechanism for H2A.X incorporation into chromatin (Fig. 8). In cells, such as HCT-116 and Jurkat, which are able to incorporate sufficient H2A.X into the newly synthesised chromatin during DNA replication, due to higher H2A.X SL mRNA levels, do not require de novo H2A.X incorporation for an efficient DNA damage response. In contrast, cells including HeLa and RPE-1 with lower levels of H2A.X SL mRNA display lower levels of steady state chromatin H2A.X. Consequently, upon DNA damage de novo H2A.X is immediately synthesised from pre-existing H2A.X poly(A) mRNA and incorporated into the chromatin, to efficiently signal the DNA damage.

## Discussion

The H2A.X protein has been extensively studied as a marker of DNA damage repair. However, how *H2AFX* expression is regulated through its two mRNA isoforms and what role these may play in the DNA damage response remains unknown. The aim of our study was therefore to investigate the role of these two H2A.X mRNA isoforms in the DNA damage response. It was previously observed that the H2A.X poly(A)/SL mRNA ratio significantly varies between cell lines^24^. Here we have we compared cell lines that differentially express the two H2A.X mRNA isoforms; HeLa and RPE-1 cells with predominantly H2A.X poly(A) mRNA *versus* HCT-116 and Jurkat with mainly H2A.X SL mRNA (Fig. 2).

As summarised in Fig. 8, we show that HCT-116 and Jurkat cells contain larger amounts of H2A.X protein in their chromatin, resulting in a lower dependence on the H2A.X poly(A) mRNA for an efficient DNA damage response. Increased levels of S-phase-specific H2A.X SL mRNA allow for higher H2A.X deposition on chromatin during DNA replication. This results in a larger H2A.X/H2A protein ratio in chromatin, which is sufficient for efficient γH2A.X signalling by phosphorylation of the chromatin H2A.X, both inside and outside of the S-phase. In contrast, HeLa and RPE-1 cells have lower amounts of H2A.X protein deposited in chromatin during DNA replication. Upon DNA damage induction, these cells require de novo synthesis and chromatin deposition of H2A.X from the H2A.X poly(A) mRNA isoform. This newly incorporated H2A.X in chromatin is required for sufficient H2A.X phosphorylation to form γH2A.X foci. As H2A.X poly(A) mRNA levels do not change in response to DNA damage, H2A.X protein levels must be regulated through activation of translation of the mRNA and/or an increase in protein stability. In support of the latter possibility, it has been shown that under normal conditions H2A.X protein is constantly degraded by poly-ubiquitination^45^ and thus not incorporated into chromatin, whereas DNA damage induction leads to the stabilisation of the H2A.X protein allowing incorporation into chromatin to form γH2A.X foci. As we could only detect H2A.X protein in chromatin fractions but not in cytoplasmic or nucleoplasmic fractions (Supplementary Fig. 4a) we postulate that stable de novo H2A.X is immediately deposited onto chromatin.

How cells regulate different amounts of the H2A.X SL and poly(A) mRNA remains unresolved. We therefore manipulated HCT-116 cells that normally express large amounts of SL RNA in S-phase, to prevent processing at the SL by deleting the HDE and so abolish production of H2A.X SL mRNA. Surprisingly, this did not lead to increased H2A.X poly(A) mRNA levels in the S-phase (Supplementary Fig. 7b). In effect we predict that a regulatory mechanism exists in the S-phase to prevent processing at the downstream poly(A) site even if processing at the SL-HDE site is restricted. Similarly, we were unsuccessful in increasing the amount of H2A.X SL mRNA in HeLa cells by introducing an H2A.X transgene that had the poly(A) site removed (Supplementary Fig. 6b). While this transgene did not produce any poly(A) RNA, it produced no more SL RNA than a wild-type H2A.X transgene integrated at the same site. The fact that H2A.X SL mRNA levels did not increase implies that SL mRNA 3’-end processing is an intrinsic feature of this gene in HeLa cells.

A possible explanation for the different H2A.X mRNA isoform ratios in different cell lines may relate to U7 snRNA expression levels. Thus HCT-116 cells, which express high amounts of H2A.X SL mRNA, also express increased U7 snRNA levels (Supplementary Fig. 3a). To directly investigate this possibility, we overexpressed U7 snRNA in HeLa cells by co-expressing the proteins LSM10 and LSM11, which results in a two-fold increase in U7 snRNA levels due to the higher efficiency of U7 snRNP packaging (Supplementary Fig. 3b). However, the overexpression of U7 snRNP alone did not increase H2A.X SL mRNA levels in HeLa cells (Supplementary Fig. 3c). This does not exclude the possibility that U7 snRNA levels play a role in the regulation of H2A.X mRNA isoform ratios. However, it does indicate that higher U7 snRNA levels alone are insufficient. It is likely that other factors that bind the H2A.X pre-mRNA and/or transcription factors binding to the H2A.X promoter play a role in regulating H2A.X mRNA ratios. Overall it appears that the levels of H2A.X SL mRNA expression are maintained in cells by multiple mechanisms. In effect these levels are “hardwired” features of particular cell types.

It is apparent that different cell types have evolved distinct approaches to provide sufficient H2A.X to satisfy the needs of the DNA damage response. Rapidly dividing cells likely produce sufficient H2A.X from the S-phase-specific SL mRNA. In contrast, other cell types have elected to employ the more widely expressed poly(A) mRNA to achieve a full DNA damage response.

## Methods

### Cell culture and transfections

RPE-1 cells were kindly provided by Jean Cook (University of North Carolina at Chapel Hill), parental HeLa Flp-In T-REx cells by Matthias Gromeier (Duke University), HCT-116 cells by the Marzluff lab and Jurkat J76 cells by Oreste Acuto (University of Oxford).

HeLa and RPE-1 cells were maintained in high glucose Dulbecco’s Modified Eagle’s Medium (DMEM) and HCT-116 cells in McCoy’s 5a Medium, both supplemented with 10% foetal bovine serum (FBS), 100 Units/mL penicillin and 100 µg/mL streptomycin. Jurkat J76 cells were grown in RPMI-1640 supplemented with 10% FBS.

The human iPSC lines used have been described previously (SFC840-03-03 PMID:26905200, SFC841-03-01 PMID:27097283; and SFC856-03-04 PMID:28827786), and are available through https://ebisc.org/. They were derived from healthy donors recruited through StemBANCC/Oxford Parkinson’s Disease Center: participants were recruited to this study having given signed informed consent, which included derivation of hiPSC lines from skin biopsies (Ethics Committee: National Health Service, Health Research Authority, NRES Committee South Central, Berkshire, UK [REC 10/H0505/71]). They were cultured at the James Martin Stem Cell Facility, University of Oxford, in feeder-free culture on geltrex in E8 medium (both Thermo Fisher).

HeLa and HCT-116 cells were transfected with siRNAs using Lipofectamine RNAiMAX (Invitrogen, #13778-150) and RPE-1 cells using Dharmafect 1 Transfection Reagent (Dharmacon, #T-2001-01), according to manufacturer instructions. SiRNA sequences are listed in Supplementary Table 2. Plasmids were transfected using X-tremeGENE 9 (Sigma, # 6365787001) according to manufacturer instructions. To induce DNA damage, cells were treated with 5 µM etoposide (Sigma, # E1383) for 2 hr.

### Cell cycle synchronisation

Nocodazole and aphidicolin blocks were performed as decribed^46^. To synchronise HeLa cells by double thymidine block (DTB), 2 mM thymidine (Sigma, #T1895) was added and incubated for 19 hr. Cells were released from the first thymidine block by washing twice with PBS followed by the addition of fresh culture media. After 9 hr, 2 mM thymidine was added and incubated for 18 hr. After the release from the second block time points were collected. For DTB synchronisations of HCT-116 cells, 2 mM thymidine was added for 12 hr, then released and regrown for 9 hr, followed by a second block of 12 hr.

RPE-1 cells were synchronised by contact inhibition as described^37^. Cells were seeded to be subconfluent 24 hr later. For RNAi experiments, the cells were transfected at the subconfluent state. The day after the transfection, the cells were 100% confluent and then incubated for a total of 4 days. During this time the cells undergo contact inhibition entering G0 arrest. The cells were then split 1/10 into a new cell culture and harvested at different time points.

### Total RNA and library preparation for RNA sequencing

Total RNA was extracted using Tri-Reagent (Sigma, #T9424) according to manufacturer instructions. Prior to library preparation, ribosomal RNA (rRNA) was depleted using the Ribo-Zero Magnetic Kit (Human/Mouse/Rat) from Illumina (#MRZH116). 500 ng ribo-depleted RNA was employed for library preparation with the NEBNext Ultra Directional RNA Library Prep Kit (Illumina, #E7420S). Libraries were analysed on a bioanalyser (Agilent 2100 Bioanalyzer system) using the Agilent High Sensitivity DNA Kit (Agilent, #5067-4626). 50 bp paired-end sequencing was performed by the Wellcome Trust Centre for Human Genetics (University of Oxford, UK).

### RNA-sequencing data processing

Adaptors from the RNA-sequencing reads were trimmed using Cutadapt 1.8.3 and reads were mapped using Tophat 2.0.13 (with parameters -g 1 -r 3000 -no-coverage-search) to the GRCh38/hg38 reference genome. Only correctly paired and properly mapped reads were selected using SAMtools 0.1.19. The data were visualised using Bedtools 2.23.0 and scaled to the size of each library (genomeCoverageBed). The UCSC genome browser was employed to visualise the data. Note that although sequencing reads were normalised to library size, RNA level differences are only qualitative in nature, due to the various fractionation steps.

Coordinates of all annotated histone transcripts were extracted from GRCh38 Gencode v27 (Ensembl 90). Stem-loop locations were identified for any transcript (also multiple ones per gene) in regions from CDS start to (CDS end + 600 bp) by finding the following sequences: GGCTCTTTTCAGAGCC, GGCCCTTTTCAGGGCC, GGTTCTTTTCAGAGCC, GGTTCAAAAGAGAGCT, GGCCCTTTTAAGGGCC, CGGCTCTTTTCAGGGCC, GGCCCTTTTTAGGGCC, GGCTCTTTTAAGAGCC, GGCTCTTTTTAGAGCC, GGCCCTTCTCAGGGCC, GGCTCTTCTAAGAGCC, GGCTCTTCTCAGAGCC. All these sequences were located downstream of the CDS end. Histone genes with multiple isoforms (including spliced) were checked for evidence from the sequencing data, that the stem-loop of the longer/spliced isoform was used. If UTRs were annotated in Gencode, then regions from the CDS end to the UTR end + 2 kb (in transcriptional direction) were scanned for PAS signals (AATAAA > ATTAAA > ATGAAA > TATAAA), otherwise the scan was performed for CDS end to CDS end + 2 kb. These PAS were used as indicative to check histone genes manually for usage of the PAS based on abrupt signal loss in poly(A)+ sequencing data. If the signal loss was not in the CDS end to CDS end + 2 kb region, but further downstream, that region was checked for a possible PAS.

### Fractionation of nuclear and cytoplasmic RNA or protein

Cells were resuspended in HLB+N buffer (10 mM Tris-HCl pH 7.5, 10 mM NaCl, 2.5 M MgCl_2_ and 0.5% NP-40), incubated on ice for 3–10 min and pelleted at 300 x *g* for 3 min at 4°C. Cytoplasmic lysates were transferred to a new tube and either Tri-Reagent (Sigma, #T9424) was added for RNA isolation or the lysate was directly employed for protein quantification. The nuclear pellet was resuspended once more in HLB+N buffer and carefully overlayed on 500 μL HLB+NS buffer (HLB + N buffer with 10% sucrose). After pelleting the nuclei at 300 x *g* for 10 min at 4°C, the SN was removed and the nuclear pellet was lysed either in Tri-Reagent for RNA extraction or in RIPA buffer (made from 10x RIPA: 0.5 M Tris-HCl pH 7.4, 1.5 M NaCl, 2.5% sodium deoxycholate, 10% NP-40, 10 mM EDTA) for protein extraction.

### Chromatin fractionation protocol

Cells were fractionated in cytoplasmic and nuclear fractions as described above. The nuclear pellet was resuspended in NUN1 buffer (20 mM Tris-HCl pH7.9, 75 mM NaCl, 0.5 mM EDTA pH 8.0, 50% Glycerol). Then NUN2 buffer (20 mM HEPES pH 7.6, 7.5 mM MgCl_2_, 0.2 mM EDTA pH 8.0, 0.3 M NaCl, 1 M Urea, 1% NP-40) was added, followed by a 15 min incubation on ice and vortexing every 3–4 min. Chromatin was pelleted at max. speed for 2 min at 4°C. The supernatant was kept as nucleoplasmic fraction. The pellet was washed with PBS and resuspended in PBS supplemented with benzonase (2 µL per 100 µL of PBS, Sigma, # E1014), digested at 37°C for 15 min, then 4x protein loading dye (400 mM Tris-HCl pH 6.8, 10% SDS w/v, 40% Glycerol, 4 mM EDTA, 400 mM DTT and 0.1% bromophenol blue) was added to a final concentration of 1x and the lysate was incubated at 95°C for 15 min. The resulting chromatin lysate was diluted 1/5 with 1x protein loading dye and 5–10 µL were used for western blot.

### Reverse transcription and PCR

Complementary DNA (cDNA) was made using random hexamers (Life Technologies, #48190011) and SuperScript IV Reverse Transcriptase (Life Technologies, #18090050), according to manufacturer instructions. PCR was performed with Phusion polymerase (NEB, #M0530S) and 10% 5 M Betaine (Sigma, #61962). Real-time PCR was performed with SensiMix (Bioline, #QT650-05) according to manufacturer instructions.

### Northern blot

For H2A.X mRNA detection, RNA was separated on 1% agarose containing 5.5% v/v formaldehyde 36.5–38% in MOPS and transferred by capillarity in 10x SSC (1.5 M NaCl and 150 mM tri-sodium citrate). For U7 snRNA northern blots, RNA was separated on 8% polyacrylamide gels (60% v/v 1 x TBE containing 7 M Urea, 40% v/v 19:1 acrylamide:bisacrylamide 20%, 0.1% ammonium persulfate, 0.01% TEMED) in TBE followed by semi-dry transfer. HIST1H4A and EEF1A1 mRNA was detected with dsDNA probes, generated by PCR (primers listed in Supplementary Table 2), radioactively labelled with ^32^P-α-dCTP (PerkinElmer, #NEG51250UC) using the Prime-IT II Random Primer Labeling Kit (Agilent, #300385), as described by the manufacturer’s instructions. To detect the two H2A.X mRNA forms, a ssDNA probe was designed and ordered as custom DNA oligo with Integrated DNA Technologies (IDT) (Supplementary Table 2). This probe was end-labelled with ^32^P-γ-ATP (PerkinElmer, #NEG502Z250UC) using T4 polynucleotyde kinase (NEB, #M0236S). The labelled probes (dsDNA and ssDNA) were purified using Illustra Microspin G-25 columns (GE Healthcare Life Sciences, #27-5325-01), as described by the manufacturer’s instructions, and denatured at 85°C for 3 min prior addition to the tube containing the membrane and the hybridisation buffer (Sigma, #H7033). Probe hybridisation was performed for 16 hr at 65°C, rotating in a hybridisation oven.

The probes for U6 and U7 snRNA were generated by in vitro transcription using the MAXIscript SP6/T7 Transcription Kit (Thermo Fisher Scientific, #AM1320) and ^32^P-αCTP (PerkinElmer, #NEG508H250UC). The U6 and U7 snRNA sequences followed by the bacteriophage SP6 promoter sequence were cloned downstream of the T7 promoter of the pCI vector (Promega, #E1731). PCR products using T7 and SP6 forward primers were used as template for in vitro transcription. The antisense snRNA sequences generated with SP6 RNA polymerase were used as probes for northern blotting.

### Western blot for histone protein detection

To detect total histone protein levels by western blot, the cell pellet was directly lysed in 4x SDS loading dye (400 mM Tris-HCl pH 6.8, 10% SDS w/v, 40% Glycerol, 4 mM EDTA, 400 mM DTT and 0.1% bromophenol blue) and incubated at 95°C for 15 min. The samples were diluted to 1x SDS loading dye and separated on 12% Bis-Tris Protein Gels (Life Technologies, #NP0342BOX) with NuPAGE MES SDS Running Buffer (Life Technologies, #NP0002). Antibodies are listed in Supplementary Table 3. All western blots shown in figures are representative of at least 3 independent experiments. Quantification by densitometry was performed using Image Studio Lite software.

### Immunofluorescence (IF)

#### Staining

EdU labelling was performed using the Click-iT EdU Alexa Fluor 647 Imaging Kit (Thermo Fisher Scientific, #C10340), according to manufacturer’s instructions. The primary antibody (γH2A.X, Milipore, #05-636) diluted 1:200 in complete tissue culture media was incubated for 2 hr at room temperature. After 3x washes with PBS 0.01% Tween-20, the secondary antibody (Alexa Fluor 594, Life Technologies, #A11032) diluted 1:1000 in complete media was applied and incubated for 1 hr at room temperature. Following 3x more washes with PBS 0.01% Tween-20, 1:1000 DAPI staining solution was applied (Thermo Fisher Scientific, #62248). Finally, the cover slips were washed twice with water and mounted on a slide using ProLong Gold Antifade Reagent (Thermo Fisher Scientific, #P36930).

#### Image acquisition

IF imaging was performed with an Olympus FV1200 confocal laser scanning microscope. The system was equipped with a 60x/1.42-NA lens, standard photomultiplier (PMT) detectors, 655–755 nm filter (BA655-755), SDM640 and SDM490 dichroic mirrors and 405 nm, 559 nm and 635 nm lasers. Settings were adjusted to the sample with the brightest signal. All the samples from the same experiment were imaged with these same settings to be able to compare intensities between samples. Of each slide, z-stacks of 10 different fields were imaged.

#### Image analysis

The microscopy images were processed in the Image J software (version 2.0.0).

### Flow cytometry

Propidium iodide staining^46^ and EdU labelling followed by staining with Alexa647-azide and DAPI^37^ were performed as described. Samples stained with propidium iodide were analysed with the BD FACS Calibur with the 488 nm laser using a 585/42 band pass filter. Samples labelled with EdU (Alexa 647) and stained with DAPI were analysed with BD LSRFortessa X-20. DAPI was detected with the 405 nm laser, a 450/50 band pass filter and a 420 nm long pass filter. Alexa 647 was detected using a 640 nm laser, a 670/30 band pass filter and a 665 nm long pass filter.

### CRISPR-Cas9 gene editing

The CRISPR-Cas9 guide RNA (gRNA) as well as the template for homologous recombination were designed by Joey Riepsaame (University of Oxford). Target specificity of the gRNA was analysed using the CCTop online Tool (https://crispr.cos.uni-heidelberg.de). Two gRNAs were designed to cut around the *H2AFX* HDE (Supplementary Table 2). The symmetric single-stranded donor oligonucleotide (ssODN) employed as template for homologous recombination was designed in the antisense direction of the gene, with a 26 bp left homology arm and a 29 bp right homology arm and deleting nucleotides 574–598 of the *H2AFX* gene (counting from the first nucleotide of the 5’ UTR) (Supplementary Table 2). An EcoRI restriction site was inserted between the homology arms, to facilitate the screening for positive clones.

The gRNAs were in vitro-transcribed as described^47^ and assembled with recombinant Cas9 protein. For RNP formation, 1 μg of each gRNA was added to 5 μg (HiFi) Cas9 protein (IDT, #1081060) in a total volume of 20 μL IDT Duplex Buffer (IDT, #11-05-01-12), mixed by pipetting up and down for 5 times and incubated at 37°C for 5 min. Resulting RNPs were transfected into the cells together with 100 pmol of the template for homologous recombination by electroporation using the NEON Transfection System (Thermo Fisher Scientific, #MPK5000), as described below.

Cells were seeded in antibiotic-free media 24 hr before transfection. 2 hr before harvesting for transfection, cells were treated with non-homologous end joining (NHEJ) inhibitor SCR-7 (Sigma, #SML1546). Cells were washed twice with PBS and nucleofected with the pre-assembled RNPs and the ssODN (described above) in a total volume of 10 μL “buffer R” (Invitrogen, #MPK1096). For HeLa cells 1005 V, 35 ms and 2 pulses were used and for HCT-116 1530 V, 20 ms and 1 pulse. After transfection, cells were immediately transferred to a 24-well plate containing 0.5 mL antibiotics-free media supplemented with 1 μM SCR-7. The day after transfection, media was changed to media with antibiotics, without SCR-7. After 48 hr, cells were split 1/10 into a new 24-well plate and remaining cells were employed to seed 1 cell per well in a 96-well plate by fluorescence-activated cell sorting (FACS) using the BD FACSAria III, each well containing 50 μL complete media. For cell sorting, DAPI was added to avoid dead cells. Cells remaining after FACS sorting were employed for genomic DNA extraction followed by bulk PCR to assess the proportion of deletion mutants *versus* WT cells. PCR screening was performed with 5’-CGGGCGTCTGTTCTAGTGTT-3’ as forward primer and 5’-ACGGAGGTCCCCGAAGAG-3’ as reverse primer, resulting in a PCR product of 792 bp in the WT and 773 bp in the deletion mutant. To distinguish the two, the PCR product was cut with EcoRI, showing two fragments (557 bp and 216 bp) only in the EcoRI-containing deletion mutants.

### Generation of HeLa Flp-In T-REx stable cell lines

#### H2A.X constructs

For stable integration of *H2AFX* gene constructs into HeLa-FlpIn cell line, the pcDNA5-FRT-TO-eGFP-Linker plasmid, kindly provided by the Castello laboratory (Oxford University), was employed as backbone. The region containing the CMV promoter up to after the bGH (bovine growth hormone) poly(A) site was excised using MluI and SphI restriction enzymes (New England Biolabs (NEB, #R0198S and #R0182S). The linearised plasmid was gel-purified using the Zymoclean Gel DNA Recovery Kit (Zymo Research, #D4007) and dephosphorylated using the FastAP Thermosensitive Alkaline Phosphatase (Thermo Fisher Scientific, #10819230). For the insert, the H2A.X gene was amplified from HeLa genomic DNA using 5’-CTATCGacgcgtTTCCCAGACGCTCTCTAGGT-3’ as forward primer, binding 335 base pairs (bp) upstream of the *H2AFX* 5’UTR, thus including the *H2AFX* promoter, and con- taining the MluI restriction site. As reverse primer, 5’-TTACATgcatgcGTTCTCCTGGTAC GTCCTTTCT-3’ was employed, binding 1807 bp downstream of the *H2AFX* 5’UTR start, including the entire *H2AFX* gene with its own poly(A) site and containing the SphI restriction site. The PCR was performed with Q5 polymerase (NEB, #M0491L), HeLa genomic DNA as template. The PCR product was gel-purified, digested with MluI and SphI, gel purified once more and ligated into the linearised and dephosphorylated plasmid. Ligation was performed using T4 DNA ligase (NEB, #M0202S). The ligated product was transformed into E. coli XL1-Blue competent cells. Positive clones were selected by colony PCR and Sanger Sequencing using primers listed in Supplementary Table 2. The resulting plasmid called pcDNA5-FRT-*H2AFX* wt was employed as a template to make *H2AFX* mutant constructs by site-directed mutagenesis.

#### LSM10/11 construct

A pcDNA3 vector containing the mouse LSm10-2A-LSm11 cassette was kindly provided by Sarah Tisdale and Livio Pellizzoni. The cassette was transferred into the pcDNA5-FRT-TO-CMV vector described above and transfected into HeLa-Flp-In T-REx cells as described below in section “Stable integration into HeLa-FlpIn T-REx cells”. To induce LSm10/11 expression, cells were treated with 1 µg/mL doxycycline for 3 days.

#### Site-directed mutagenesis

Several *H2AFX* mutants were made by site-directed mutagenesis using the pcDNA5-FRT- *H2AFX* wt plasmid as initial template. Single nucleotide point mutations were made with 100% overlapping primers, whereas for larger deletions only partially overlapping primers were designed as described^48^. PCR was performed with Q5 polymerase (NEB, #M0491L). Supplementary Table 2 shows the constructs with their corresponding mutagenesis primers, the template plasmids, the PCR melting temperatures and the deleted nucleotide positions. After DpnI (NEB, #R0176S) treatment, the plasmid was transformed in XL1-Blue *E. coli* competent cells.

#### Stable integration into HeLa-FlpIn T-REx cells

For stable integration of the construct into the HeLa-FlpIn cell line 2 μg of the FRT site-containing plasmid together with 2 μg pOGG4 Flp-recombinase Expression Vector (Thermo Fisher Scientific, #V600520) were resuspended in 200 μL Opti-MEM (Gibco, #31985-062). 12 μL X-tremeGENE 9 DNA Transfection Reagent (Sigma, #6365787001) were added and the mix incubated at room temperature for 15–30 min. During this incubation period, 3.5 × 10^6^ HeLa-FlpIn cells were seeded in a 10 cm dish, in 10 mL antibiotics-free media and the transfection mix was applied. 24 hr after transfection, the media was changed to DMEM containing antibiotics and 400 μg/mL Hygromycin B (Invitrogen, #10687010) to select positive cells. The day after, the media was changed again and only 150 μg/mL Hygromycin B were added. After about two days, the media was changed again to eliminate dead cells and the remaining cells were grown until the appearence of colonies. Cells were then genotyped, further expanded and frozen in multiple vials.

### Pulsed-field gel electrophoresis

Cells were harvested using trypsin and washed with PBS. Cells were counted using a NC-3000 Advanced Image Cytometer (Chemometec). Each sample was counted twice to increase accuracy. 0.2 × 10^6^ cells were employed to prepare agarose plugs with the CHEF Mammalian Genomic DNA Plug Kit (BioRad, #1703591), according to the manufacturer’s instructions. Pulsed-field gel electrophoresis (PFGE) was performed as previously described^49^.

### Quantification and statistical analysis

Quantifications were performed from at least 3 biological replicates unless indicated otherwise in figure legend. Statistical *P*-values were calculated by multiple *t*-tests or two-way Anova using Graphpad Prism software, Version 8.1.2. ns: *P*-value >0.05. *P-value ≤ 0.05. **P-value ≤ 0.01. ***P-value ≤ 0.001. ****P-value ≤ 0.0001. The error bars denote SD.

### Reporting summary

Further information on research design is available in the Nature Research Reporting Summary linked to this article.

## Supplementary information

Supplementary Information

Reporting Summary

## Data Availability

Genome-wide datasets are deposited at GEO under the accession number GSE144323. All data is available from the corresponding author upon reasonable request. Source data are provided with this paper.

## Source data

Source Data
