# Supplementary material for: Dual RNA 3’-end processing of H2A.X messenger RNA maintains DNA damage repair throughout the cell cycle
Source: Nat Commun. 2021 Jan 13;12:359. doi: 10.1038/s41467-020-20520-6 (PMC7807067; doi:10.1038/s41467-020-20520-6)
Supplement: Supplementary file 1 — Supplementary Information [file 41467_2020_20520_MOESM1_ESM.pdf]

Supplementary Figure 1

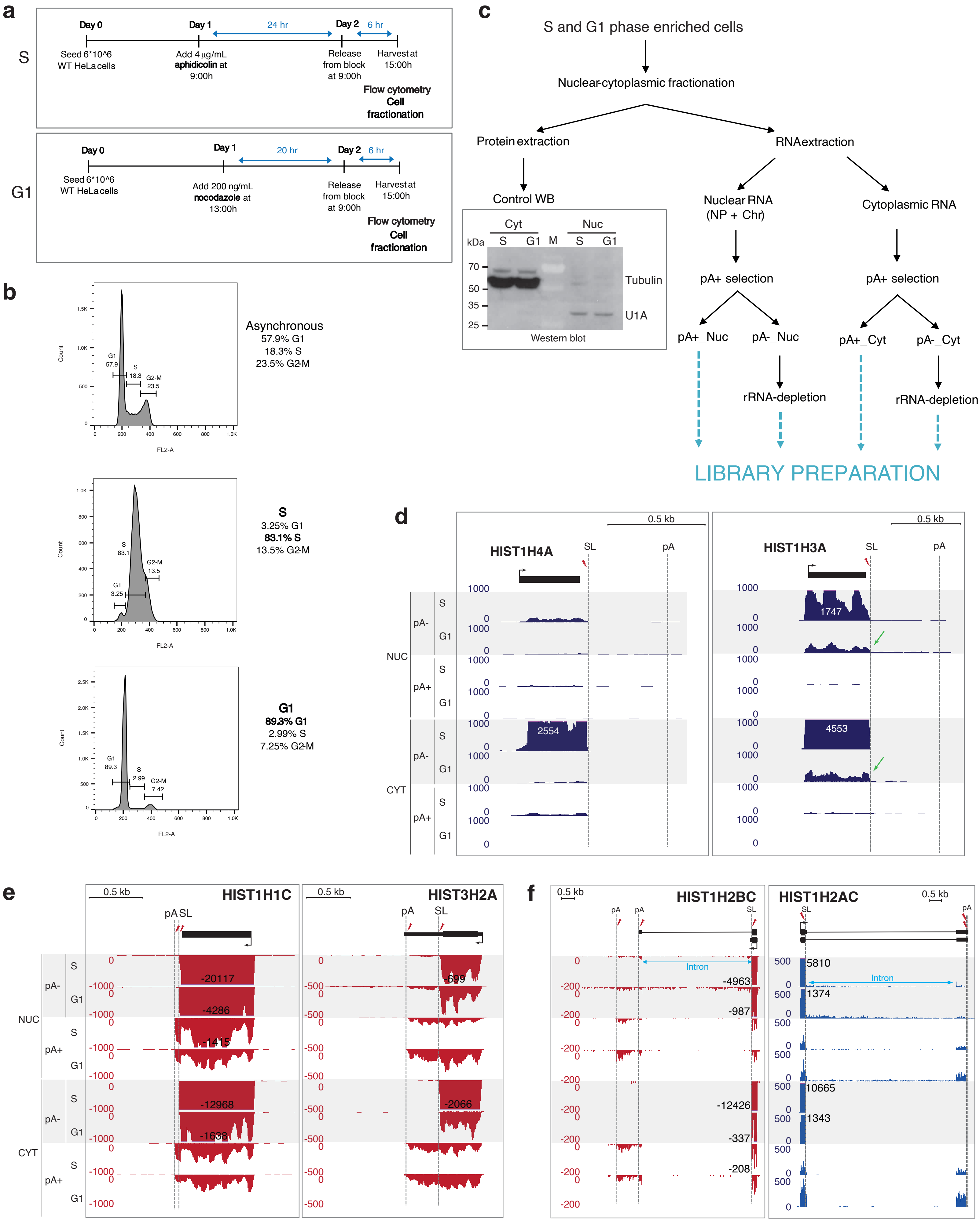

Supplementary Fig. 1 (related to Figure 1)
(a) Cell cycle synchronisation protocol.
(b) Flow cytometry analysis, showing propidium iodide fluorescence (x-axis) and the cell count (y-axis). Gating was performed manually and show percentage of cells in each cell cycle phase. Top panel shows cell cycle distribution of asynchronously growing HeLa cells as control, middle panel aphidicholin-synchronised S-phase-enriched HeLa cells and bottom panel nocodazole block G1-enriched HeLa cells.
(c) RNA fractionation steps for RNA-seq analysis. Note that nuclear RNA fraction contains nucleoplasmic (NP) as well as chromatin-bound (Chr) RNA. Fractionation efficiency was assessed by western blot with antibodies against tubulin (cytoplasmic) and spliceosomal U1A (nuclear). M: protein size marker.
(d) RNA-seq profiles of replication-dependent histone genes that generate exclusively poly(A)- SL mRNA. RNA-seq reads aligned to human genome, visualised with NCBI Genome Browser. Different tracks correspond to reads from the different fractions indicated on the left. Nuc: nuclear RNA fraction. Cyt: cytoplasmic RNA fraction. pA-: rRNA-depleted poly(A)- RNA fraction. pA+: poly(A)+ RNA fraction. S: S-phase-enriched cells. G1: G1-phase-enriched cells. SL: U7-dependent cleavage site. pA: polyadenylation site. Black arrow: direction of Pol II transcription. Green arrows: poly(A)- histone mRNA in G1 fractions most likely resulting from the remaining 3% of S-phase cells in the G1-enriched population (see percentages shown in Supplementary Fig. 1b).
(e) RNA-seq profiles of histone genes that express some poly(A)+ mRNA. Description as in (d).
(f) RNA-seq profiles of histone genes that express a fraction of intron-containing poly(A)+ mRNA. Description as in (d). We observed that the same histone genes that express polyadenylated mRNA in non-dividing tissues[20] express small amounts of polyadenylated histone mRNA in HeLa cells. Analysis of ENCODE data showed other cell lines, including human embryonic stem cells, all expressed small amounts of polyadenylated RNA from the same genes.

Supplementary Figure 2

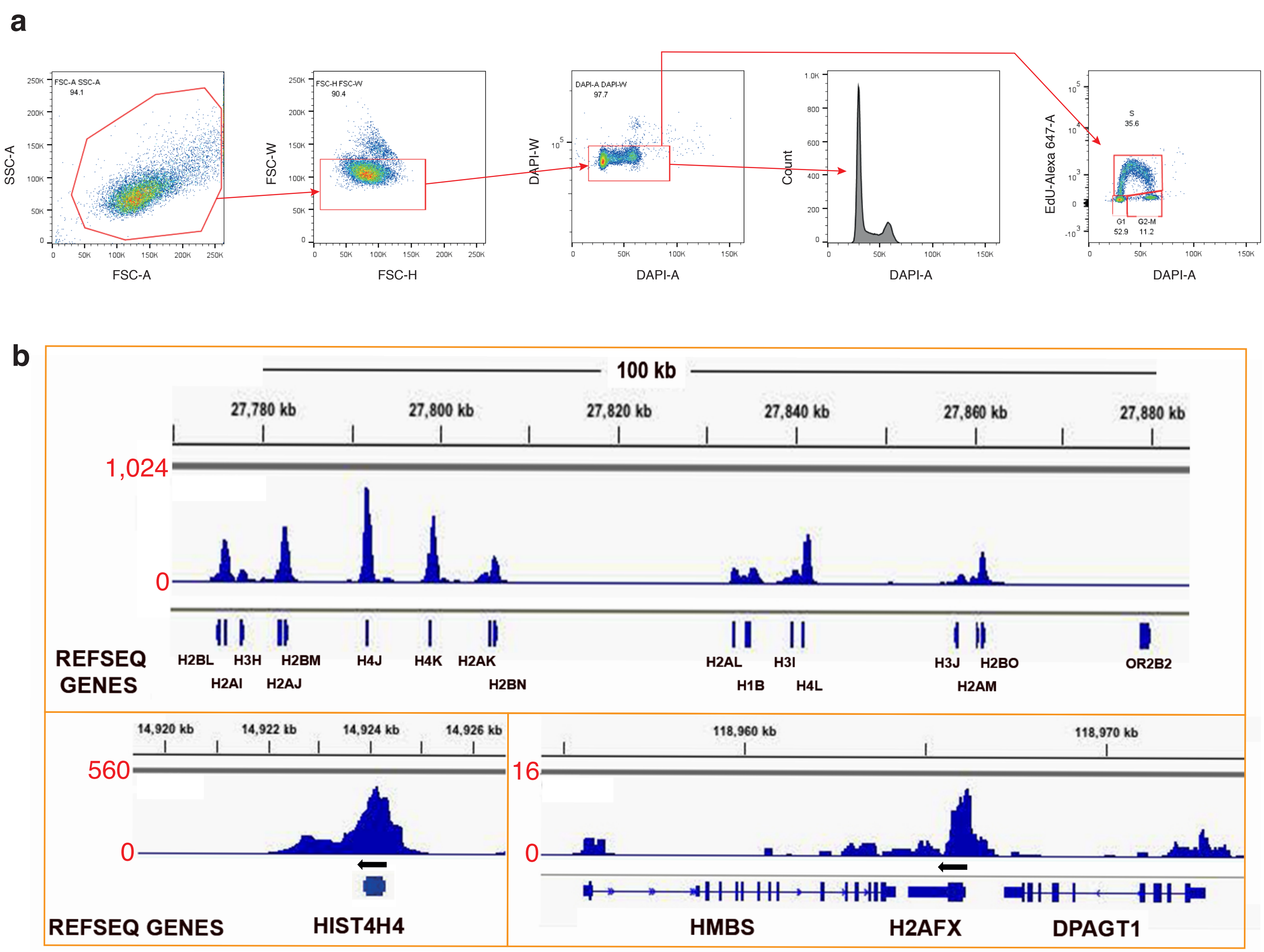

Supplementary Fig. 2 (related to Figure 2)

a) Flow cytometry gating strategy. First, living cells were separated from cell debries using a FCS-A vs SSC-A density plot. From this population, single cells were selected in a FSC-H vs FSC-W density plot. Remaining doublets were eliminated by selecting the single cell population in a DAPI-A vs DAPI-W density plot. Singlets were then plotted as a DAPI-A vs cell count histogram to visualise the cell cycle profile and as a DAPI-A vs EdU-Alexa Fluor 647-A density plot to separate S-phase cells from G1- and G2/M-phase cells.

b) Cut&Tag profiling of the RD histone gene-specific transcription factor NPAT on the RD histone genes and H2AFX in human K562 cells[42]. Scale (number of reads) indicated in red. Note strong NPAT binding to the promoters of 20 RD histone genes from a portion of histone cluster 1 on chromosome 6 (top) and to the solitary HIST4H4 gene on chromosome 12 (bottom left). In contrast, there is very low NPAT signal on H2AFX on chromosome 11 (bottom right).

Supplementary Fig. 3.

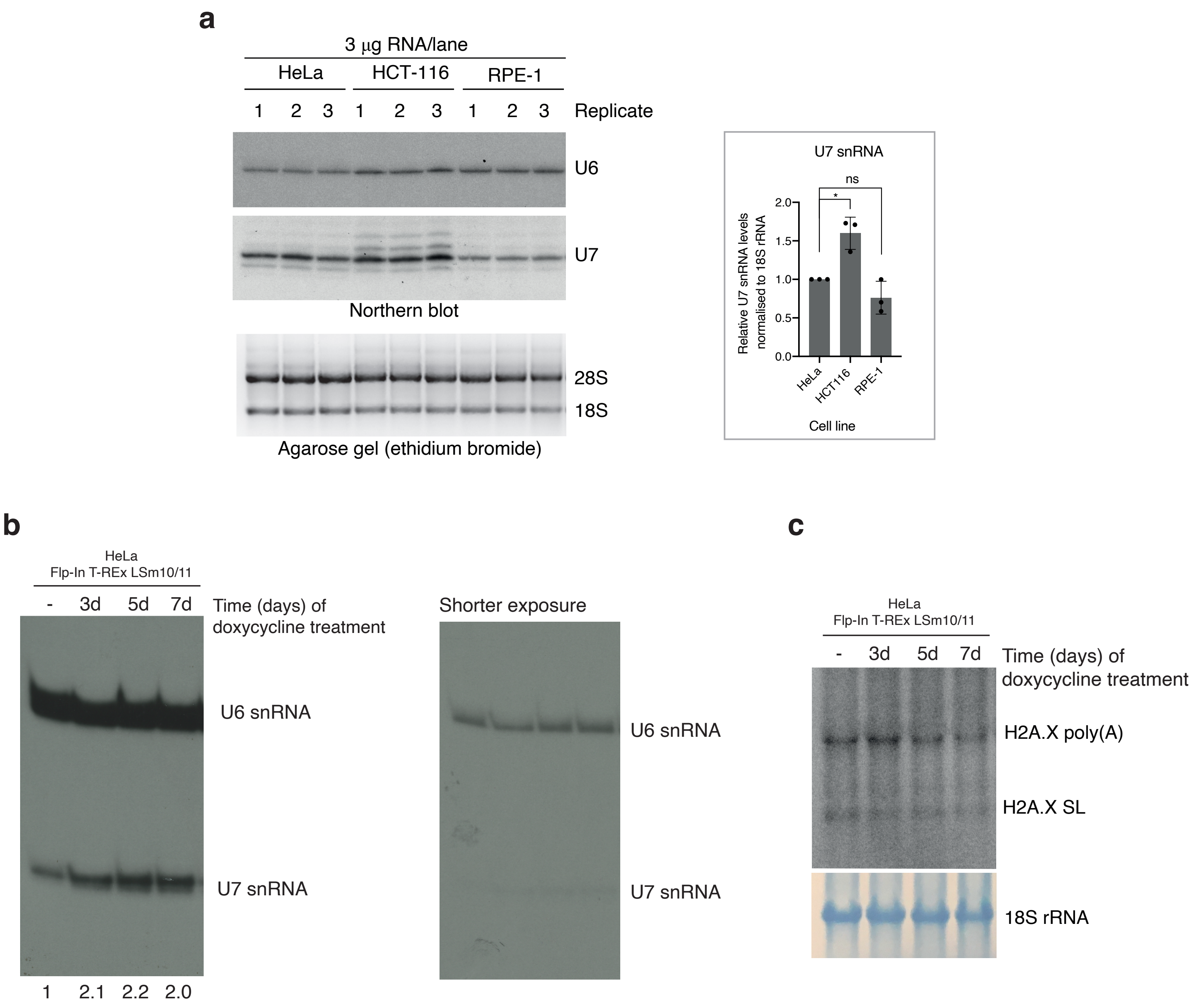

Supplementary Fig. 3 (related to Figure 3)
(a) U7 and U6 snRNA northern blot of total RNA from HeLa, HCT-116 and RPE-1 cells. Total RNA on agarose gel as loading control (bottom panel). Densitometry quantification (right panel). Mean ± SD presented for n=3. Paired Student's *t*-test, ns: not significant P-value > 0.05, *P-value ≤ 0.05.
(b) U7 snRNA northern blot of total RNA from HeLa Flp-In T-REx LSM10/11 cells treated with 1 μg/mL doxycycline for the indicated time (days). U6 snRNA was used as loading control. Shorter exposure of the same blot (right panel). Quantification by densitometry shown as fold increase following doxycycline induction.
(c) H2A.X mRNA northern blot of total RNA from samples shown in (b). 18S rRNA used as loading control.

Supplementary Figure 4

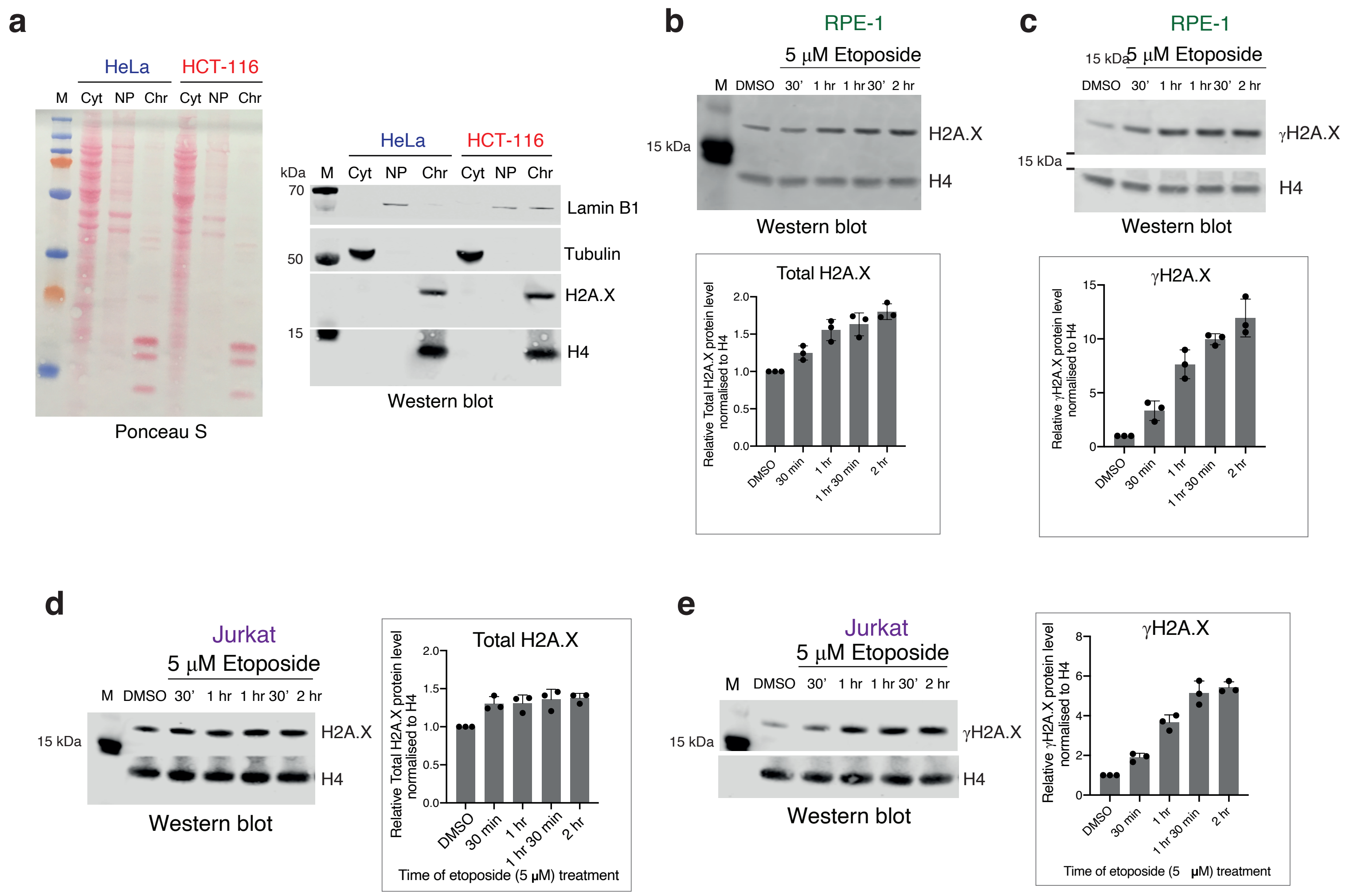

Supplementary Fig. 4 (related to Figure 4)

(a) Analysis of HeLa and HCT-116 cell fractionations by western blotting: cytoplasmic (Cyt), nucleoplasmic (NP) and chromatin (Chr) fractions. Left panel shows Ponceau S staining. Right panel shows Lamin B1 (nuclear lamina), Tubulin (cytoplasmic), total H2A.X and H4 (chromatin) levels detected using specific antibodies. M: protein size marker.

(b) Western blot analysis of H2A.X in isolated chromatin from RPE-1 cells treated with etoposide as indicated. H4 was used as loading control. M: protein size marker. Mean ± SD presented for n=3 independent experiments.

(c) As in (b) but for γH2A.X. The membrane was cut at the 15 kDa mark.

(d) Western blot analysis of H2A.X in isolated chromatin from Jurkat cells treated with etoposide as indicated. H4 was used as loading control. M: protein size marker. Mean ± SD presented for n=3 independent experiments.

(e) As in (d) but for γH2A.X.

Supplementary Figure 5

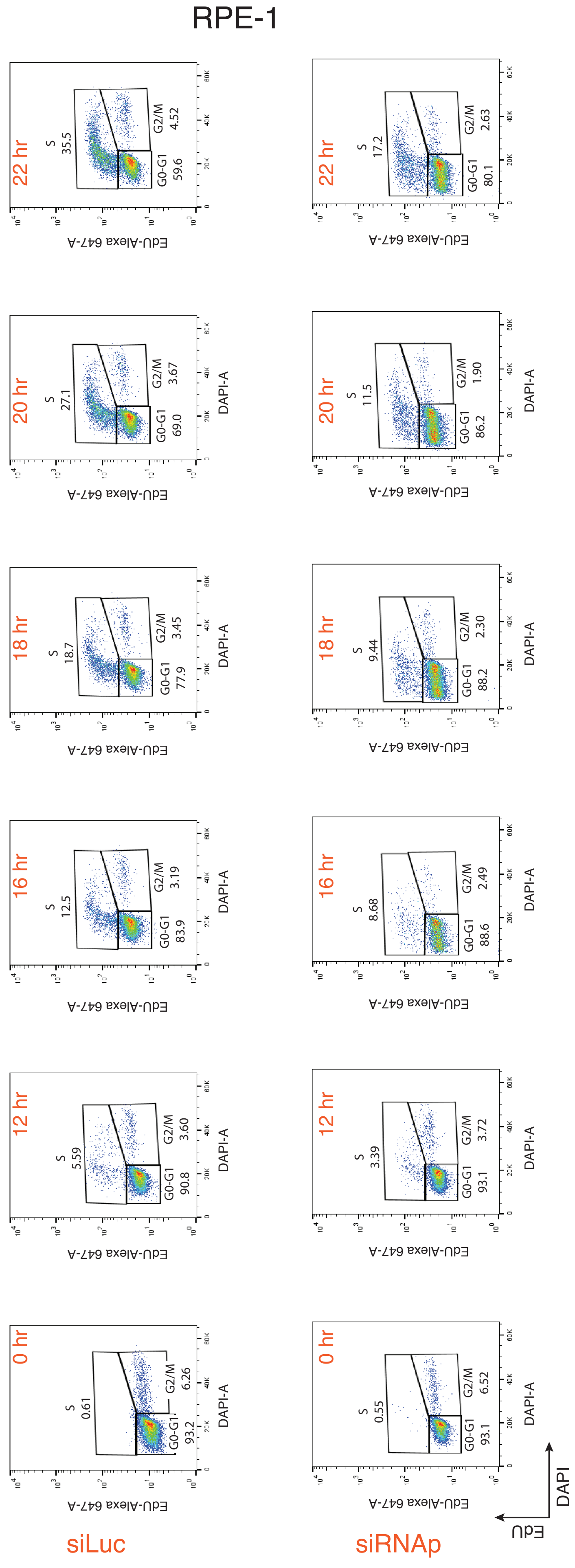

Supplementary Fig. 5 (related to Figure 5d)
Flow cytometry of EdU-labelled RPE-1 cells depleted of H2A.X poly(A) RNA and synchronised by contact inhibition. Left and right columns represent siLuc and siRNAp transfected cells, respectively. Orange numbers indicate time in hours after cell release from G0 arrest. X-axis represents fluorescent intensity of DAPI and y-axis of Alexa 647-EdU. Percentages of cells in G1, S and G2/M were analysed using FlowJo software.

Supplementary Figure 6

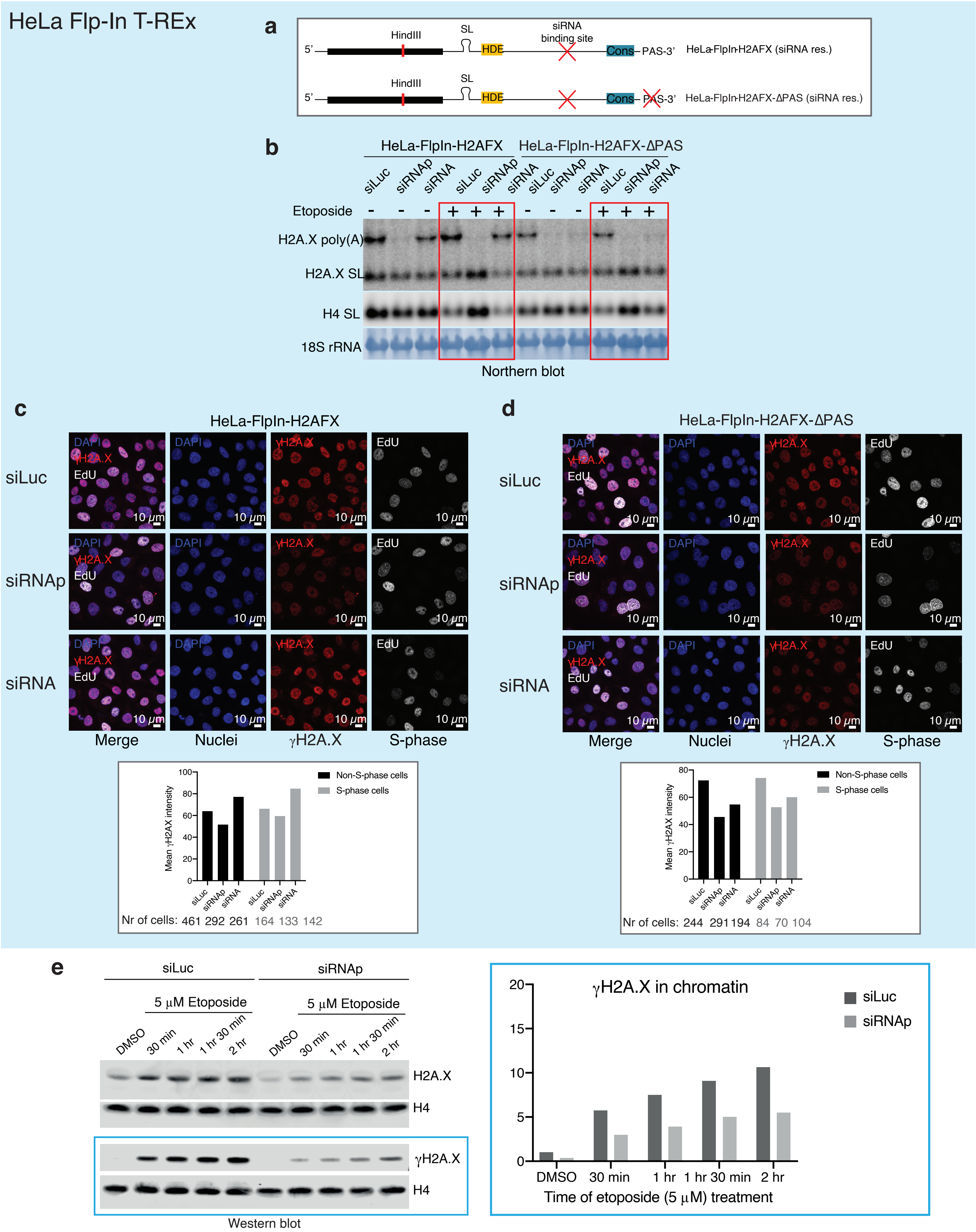

Supplementary Fig. 6 (related to Figure 6)
(a) siRNA-resistant mRNA isoforms expressed from transgenes stably introduced into HeLa-Flp-In T-REx cells. Upper transcript: siRNA-resistant WT H2AFX gene. Bottom transcript additionally lacks a 26 bp region including the PAS. The H2AFX transgene contains a HindIII site in the ORF allowing its distinction from the endogenous gene by PCR. Such PCR on genomic DNA from these cell lines followed by HindIII digestion of the PCR amplicon showed that the ratio endogenous (uncut) versus transgene (cut) was 2:1 (data not shown). As the parental HeLa cell only has one FRT site, this showed that these HeLa cells have two endogenous H2AFX genes.
(b) H2A.X mRNA northern blot of total RNA from siRNA-resistant cell lines treated with indicated siRNAs for 72 hr, without or with etoposide. H4 SL: core H4 histone SL mRNA. EEF1A1 mRNA and 18S rRNA used as loading controls. Red boxes highlight samples used for IF, shown in (c) and (d).
(c, d) IF images of siRNA-resistant HeLa-FlpIn-H2AFX (c) and HeLa-FlpIn-H2AFX-ΔPAS (d) cells. Cells were labelled with EdU (S-phase cells, white) and stained with DAPI (nuclei, blue) and anti-γH2A.X antibody by IF (red). γH2A.X quantifications in S-phase and non-Sphase cells shown below. These experiments demonstrate that the reduced γH2A.X signal is specifically caused by the loss of H2A.X poly(A) mRNA and not due to indirect effects.
(e) Western blot analysis of chromatin fraction purified from HeLa cells depleted for H2A.X poly(A) mRNA by RNAi for 72h and treated with etoposide as shown. Quantitation of these data with error bars representing standard deviation between two independent experiments. In Figure 4 we show that HeLa cells incorporate H2A.X protein into chromatin upon DNA damage. Figure 6b shows that DNA damage induction leads to increased H2A.X protein levels, suggesting that it is de novo H2A.X that is incorporated into the damaged chromatin. This experiment now shows that the H2A.X poly(A) mRNA is required for this deposition confirming that newly translated H2A.X is incorporated.

Supplementary Figure 7

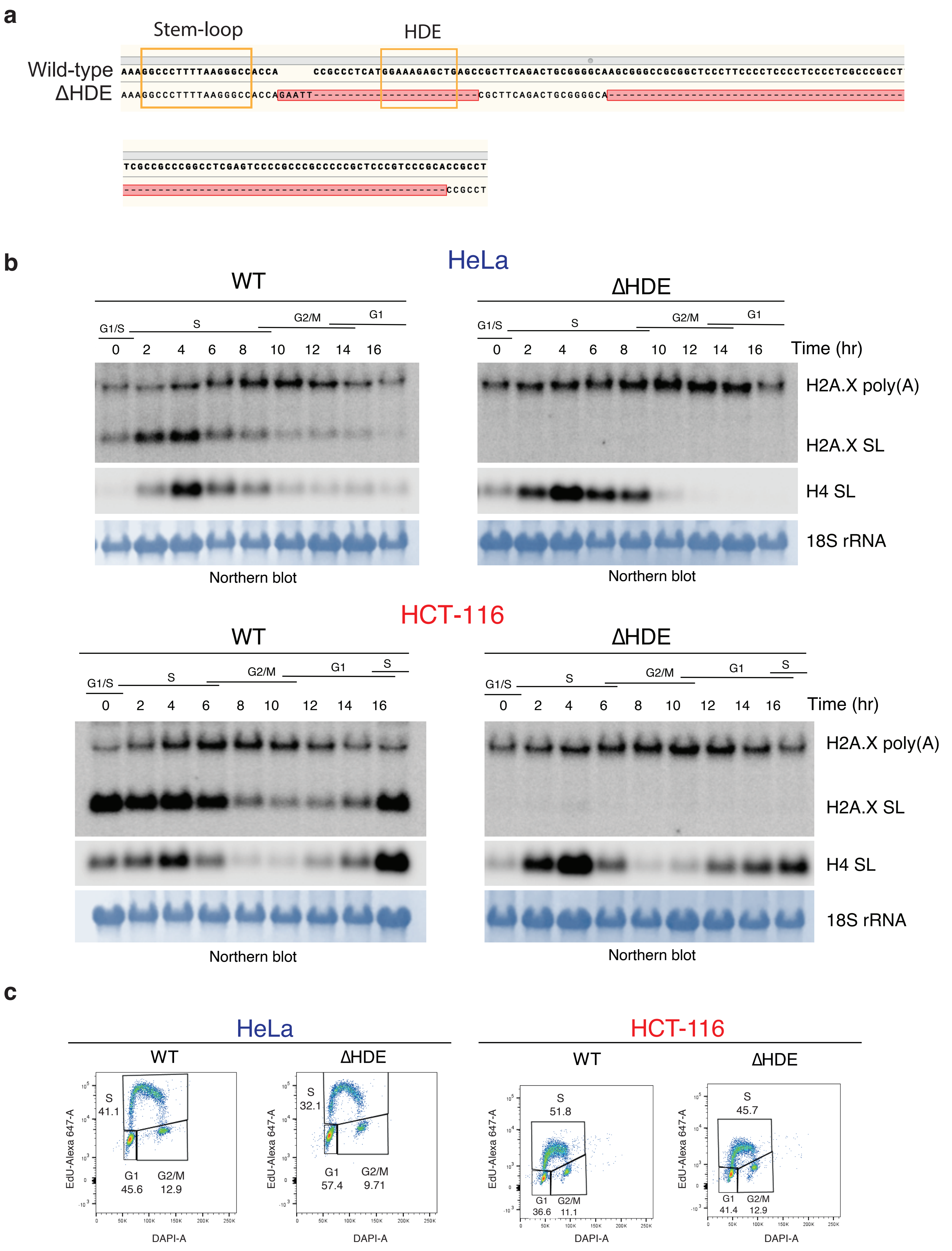

Supplementary Fig. 7 (related to Figure 7)
(a) Sequenced PCR product from genomic DNA from the HeLa ΔHDE CRISPR clone aligned to the wild-type H2A.X gene sequence using SnapGene software. A single stranded DNA oligonucleotide missing the region containing the histone downstream element (HDE) was used as template for homologous recombination. To facilitate screening for positive clones an EcoRI site (GAATTC) was instead inserted. The only PCR product obtained from the positive HeLa ΔHDE CRISPR clone also contained a 90 bp deletion downstream. Alterations to the reference sequence are highlighted in pink and deletions indicated by dashed lines.
(b) H2A.X mRNA northern blot of total RNA from DTB-synchronised WT and ΔHDE HeLa and HCT-116 cells. H4 SL: core H4 histone SL mRNA. 18S rRNA used as loading control. Time points indicate hours after release from DTB. Corresponding cell cycle phases determined by flow cytometry (data not shown) are indicated above. The deletion of the HDE abolishes the formation of the H2A.X SL mRNA because the U7 snRNA-dependent processing reaction fails to occur. As H2A.X poly(A) mRNA levels only show a mild increase in the S-phase when the HDE is deleted (specially clear in HCT-116 cells), which argues against a simple competitive effect between these two adjacent mRNA 3' end processing machineries.
(c) Flow cytometry of EdU-labelled cells. Graphs show DAPI fluorescent intensity on x-axis and Alexa 647-EdU fluorescent intensity on y-axis. Percentages of cells in G1, S and G2/M were determined using FlowJo software. HeLa cells show a 25% reduction in S-phase cells when the HDE is deleted (from 40% in WT to 30% in ΔHDE) and HCT-116 a 10% reduction (from 50% in WT to 45% in ΔHDE).

Supplementary Table 1

Gene	Strand	Chromosome	PAS coordinates
**Histone genes that only express a SL mRNA (RD histone genes)**			
HIST1H1B	-	chr6	unused
HIST1H1E	+	chr6	in tandem gene 3'UTR?
HIST1H2AD	-	chr6	unused
HIST1H2AL	+	chr6	unused
HIST1H2BB	-	chr6	chr6:26041730-26041735, unused?
HIST1H2BI	+	chr6	unused
HIST1H3C	+	chr6	unused
HIST1H3J	-	chr6	no PAS
HIST1H4A	+	chr6	unused?
HIST2H2AB	-	chr1	unused
HIST2H3D	-	chr1	unused
HIST3H3	-	chr1	unused
HIST1H4C	+	chr6	unused
HIST1H2BE	+	chr6	chr6:26184636-26184641, unused?
HIST4H4	-	chr12	chr12:14767659-14767664 (?)
HIST1H2BO	+	chr6	unknown
HIST1H3A	+	chr6	chr6:26021434-26021439
HIST1H4D	-	chr6	chr6:26186757-26186762 (?)
HIST1H1T	-	chr6	chr6:26106436-26106441
HIST1H2AB	-	chr6	chr6:26032443-26032448
HIST1H2AH	+	chr6	unknown
HIST1H2AJ	-	chr6	chr6:27813617-27813622
HIST1H2AK	-	chr6	chr6:27836022-27836027
HIST1H2BM	+	chr6	chr6:27817468-27817473
HIST1H4J	+	chr6	chr6:27825809-27825814
HIST2H3A	+	chr1	chr1:149854251-149854256
HIST2H3C	-	chr1	chr1:149839556-149839561
HIST3H2BB	+	chr1	chr1:228460308-228460313
HIST1H3F	-	chr6	chr6:26248584-26248589
HIST2H2AC	+	chr1	possibly chr1:149888153-149888158
HIST2H2BF	-	chr1	chr1:149805890-149805895
HIST1H3E	+	chr6	chr6:26227452-26227457
HIST1H2BN	+	chr6	chr6:27855557-27855562 (possibly multiple)
HIST1H3B	-	chr6	chr6:26030082-26030087
HIST1H4I	+	chr6	chr6:27140615-27140620 (?)
**Histone genes expressing poly(A)+ mRNA that is exported to the cytoplasm**			
HIST1H1C	-	chr6	chr6:26055726-26055731
HIST2H2AA3	-	chr1	chr1:149842204-149842209
HIST2H2AA4	+	chr1	chr1:149851603-149851608
HIST1H2AG	+	chr6	chr6:27135269-27135274
HIST1H2BF	+	chr6	chr6:26202679-26202684
HIST1H2BG	-	chr6	chr6:26214086-26214091
HIST1H2BL	-	chr6	chr6:27805608-27805613
HIST1H3H	+	chr6	chr6:27811278-27811283
HIST2H2BE	-	chr1	chr1:149884477-149884482
HIST2H4B	-	chr1	chr1:149860100-149860105
HIST3H2A	-	chr1	chr1:228456997-228457002
HIST1H2AI	+	chr6	chr6:27809882-27809887
HIST1H3G	-	chr6	chr6:26269421-26269426 (?)
HIST2H4A	+	chr1	chr1:149834607-149834612
HIST1H2AM	-	chr6	chr6:27891259-27891264 (?)
HIST1H2BH	+	chr6	chr6:26253689-26253694
HIST1H4E	+	chr6	chr6:26206016-26206021
HIST1H4K	-	chr6	unknown
HIST1H4B	-	chr6	unknown
**Histone genes expressing spliced poly(A)+ mRNA that is exported to the cytoplasm**			
HIST1H2BC	-	chr6	chr6:26113185-26113190
HIST1H2AC	+	chr6	chr6:26138935-26138940
HIST1H2BD	+	chr6	chr6:26171325-26171330
HIST1H2AE	+	chr6	chr6:26218348-26218353
HIST1H4H	-	chr6	chr6:26277620-26277625 / chr6:26281074-26281079
HIST1H2BJ	-	chr6	chr6:27125912-27125917 (probably further downstream)
HIST1H3D	-	chr6	chr6:26194219-26194224
HIST1H2BK	-	chr6	chr6:27138313-27138318

Supplementary Table 2

Primers used for PCR products used as templates for northern blot probes

ACTB-Fw	GGATTCCTATGTGGGCGACG	
ACTB-Rev	GTAGTCAGTCAGGTCCCGGC	
EEF1A1-Fw	TGACTGTGCTGTCCTGATTG	Designed by Silvia Ramos (University of North Carolina at Chapel Hill)
EEF1A1-Rev	CCAAAGGTGGATAGTCTGAGAA	
HIST1H4A-Fw	TGGAGAACGTGATCCGTGAC	
HIST1H4A-Rev	ATCAGCAACCTTAACCGCCA	

Primers used for real-time PCR

H2AX_pA+_Fw_EG	GGTGCTTAGCCCAGGACTTT
H2AX_pA+_Rev_EG	CCCAGCGCAGACCTATGAAT
rDNA-18S-down-fw	ATGGCCGTTCTTAGTTGGTG
rDNA-18S-down-rev	CGCTGAGCCAGTCAGTGTAG

**H2AFX northern blot probe:**	GTGGTGGCATGGGGAGGCCT<br>GGCGGCCGGCCGCGGCGCGG<br>GCCCTCTTAGTACTCCTGGGA<br>GGCCTGGGTGGCCTTCTTGCC<br>GCCCGA

Primers to amplify H2A.X poly(A) mRNA isoforms

H2A.X Total Fw-1	CGGGCGTCTGTTCTAGTGTT
H2A.X Total Rev-2	CGGGCCCTCTTAGTACTCCT
H2A.X July18 Fw-C	AGTGCTGGAGTACCTCACCG
H2A.X July18 Rev-C	GAAGGCCCGAACCCTACC

Primers to PCR the H2AX gene with its own promoter from HeLa gDNA

H2AFX_MluI_Fw_2_EG	CTATCGacgcgtTTCCCAGACGCTCTCTAGGT
H2AFX_SphI_Rev_2_EG	TTACATgcatgcGTTCTCCTGGTACGTCCTTTCT

QuikChange primers and constructs

Construct	mutagenesis primers (name)	mutagenesis primers (sequence)	plasmid used as template	Tm (°C)	Mutation
pcDNA5-FRT-TO-H2AX_HindIII	H2AX-HindIII_Quik_Fw_EG	GTCACGCCGCCCAGAAGCTTGTTGAGCTC	pcDNA5-FRT-TO-H2AX_wt	55	nt 364 G to T
	H2AX-HindIII_Quik_Rev_EG	GAGCTCAACAAGCTTCTGGGCGGCGTGAC			
pcDNA5-FRT-TO-H2AX_HindIII_dsi3	H2AFX-del-siRNA3-Fw_EG	TCAACTCGGCAAGTCGGTTAATCCCTGTCTGGACTGAGCCTCCGTTGGCT	pcDNA5-FRT-TO-H2AX_HindIII	55	1430-1454
	H2AFX-del-siRNA3-Rev_EG	CCGACTTGCCGAGTTGAGTTTGCTGGAAGGGAAATGGGGCGGCG_			
pcDNA5-FRT-TO-H2AX_HindIII_dsi3_dPAS	H2AFX-dPAS-Fw_EG	ATGGACTAAAAATGGAGCGTTTGTTTTCATTTTGCAAGAGAGTTGTAGAATTAGTGAC	pcDNA5-FRT-TO-H2AX_HindIII_dsi3	55	1430-1454 and 1566-1592
	H2AFX-dPAS-Rev_EG	GCTCCATTTTTAGTCCATCTAAAACTCCCCAATGCCTAAGGTTCTAG			

Primers for sequencing the Flp-In plasmids

Seq Primer 1-Fw	AAGCCAGTATCTGCTCCCTG
Seq Primer 2-Fw	CGGGTATTGGAGAAAAGAGCC
Seq Primer 3-Fw	CGGCAGTGCTGGAGTACC
Seq Primer 4-Fw	TGCTGCCCAAGAAGACCAG
Seq Primer 5-Fw	GTAGGGTTCGGGCCTTCC
Seq Primer 6-Fw	CAGGCCTTTCACATCAGCTC
Seq Primer 7-Fw	CTCAACTCGGCAATCCAAGC
Seq Primer 1-Rev	CTTAATGCGCCGCTACAGG

CRISPR oligonucleotides

Guide RNA 1	taatacgactcactatagGGCGGTGGTGGCCCTTAAAAgttttagagctagaaatagcaa	(protospacer sequence in capital letters)
Guide RNA 2	taatacgactcactataggCTGAGCCGCTTCAGACTGCGgttttagagctagaaatagca	(protospacer sequence in capital letters)
ssODN:	G*C*GGCCCGCTTGCCCCGCAGTCTGAAGCGAATTCTGGTGGCCCTTAAAAGGGCCTTTG*T*G	(*denote phosphorothioate bonds)

siRNA	Company	Sequence or supplier ID
siLuc (custom siRNA)	Sigma	Sense sequence (5′-3′) : GAUUAUGUCCGGUUAUGUAUU<br>Antisense sequence (5′-3′): [phos]UACAUAACCGGACAUAAUCUU
H2A.X siRNA pool (siRNAp)	Dharmacon	L-011682-00-0005
H2A.X Single siRNA (siRNA)	Integrated DNA Technologies	Sense sequence (5′-3′) : CAAUCCAAGCACCUAGAUACCAGCA<br>Antisense sequence (5′-3′): UGCUGGUAUCUAGGUGCUUGGAUUGCC

Supplementary Table 3

Antibodies for WB

**Primary**		
Actin	Sigma	Cat#A2066, RRID: AB_476693
Tubulin	Sigma	Cat#T5168, RRID: AB_477579
Total H2A.X	Bethyl	Cat#A300-082A, RRID: AB_203287
YH2A.X S139 Clone JBW301	Milipore	Cat#05-636, RRID: AB_309864
H4	abcam	Cat#ab17036, RRID: AB_1209245
U1A	abcam	Cat#ab166890
Lamin B1	abcam	Cat#ab8982, RRID: AB_1640627
**Secondary**		
IRDye 800CW	Life Technologies	Cat#926-32211, RRID: AB_621843
IRDye 680RD	Life Technologies	Cat#926-68070, RRID: AB_10956588
