# Supplementary material for: Dual RNA 3’-end processing of H2A.X messenger RNA maintains DNA damage repair throughout the cell cycle
Source: Nat Commun. 2021 Jan 13;12:359. doi: 10.1038/s41467-020-20520-6 (PMC7807067; doi:10.1038/s41467-020-20520-6)
Supplement: Supplementary file 2 — Reporting Summary [file 41467_2020_20520_MOESM2_ESM.pdf]

nature research

Corresponding author(s): Nicholas Proudfoot

Last updated by author(s): Nov 24, 2020

Reporting Summary

Nature Research wishes to improve the reproducibility of the work that we publish. This form provides structure for consistency and transparency in reporting. For further information on Nature Research policies, see our Editorial Policies and the Editorial Policy Checklist.

Statistics

For all statistical analyses, confirm that the following items are present in the figure legend, table legend, main text, or Methods section.

n/a	Confirmed	
☐	☒	The exact sample size (n) for each experimental group/condition, given as a discrete number and unit of measurement
☐	☒	A statement on whether measurements were taken from distinct samples or whether the same sample was measured repeatedly
☐	☒	The statistical test(s) used AND whether they are one- or two-sided<br>*Only common tests should be described solely by name; describe more complex techniques in the Methods section.*
☒	☐	A description of all covariates tested
☒	☐	A description of any assumptions or corrections, such as tests of normality and adjustment for multiple comparisons
☐	☒	A full description of the statistical parameters including central tendency (e.g. means) or other basic estimates (e.g. regression coefficient) AND variation (e.g. standard deviation) or associated estimates of uncertainty (e.g. confidence intervals)
☐	☒	For null hypothesis testing, the test statistic (e.g. F, t, r) with confidence intervals, effect sizes, degrees of freedom and P value noted<br>*Give P values as exact values whenever suitable.*
☒	☐	For Bayesian analysis, information on the choice of priors and Markov chain Monte Carlo settings
☒	☐	For hierarchical and complex designs, identification of the appropriate level for tests and full reporting of outcomes
☒	☐	Estimates of effect sizes (e.g. Cohen's d, Pearson's r), indicating how they were calculated

Our web collection on statistics for biologists contains articles on many of the points above.

Software and code

Policy information about availability of computer code

Data collection	No software was used
Data analysis	Adapter trimming with Cutadapt v. 1.8.3 in paired end mode with minimum-length 10 Alignment with Tophat v. 2.0.13 and the parameters -g 1 -r 3000 --no-coverage-search to hg38<br>Extraction of properly paired, properly mapped reads (samflags 0x63, 0x93, 0x53, 0xA3) with SAMtools v. 0.1.19 Data was visualized with Bedtools v. 2.23.0 (genomeCoverageBed).<br>Genome build hg38

For manuscripts utilizing custom algorithms or software that are central to the research but not yet described in published literature, software must be made available to editors and reviewers. We strongly encourage code deposition in a community repository (e.g. GitHub). See the Nature Research guidelines for submitting code & software for further information.

Data

Policy information about availability of data

All manuscripts must include a data availability statement. This statement should provide the following information, where applicable:

- Accession codes, unique identifiers, or web links for publicly available datasets
- A list of figures that have associated raw data
- A description of any restrictions on data availability

https://www.ncbi.nlm.nih.gov/geo/query/acc.cgi?acc=GSE144323

Field-specific reporting

Please select the one below that is the best fit for your research. If you are not sure, read the appropriate sections before making your selection.

☒ Life sciences ☐ Behavioural & social sciences ☐ Ecological, evolutionary & environmental sciences

For a reference copy of the document with all sections, see nature.com/documents/nr-reporting-summary-flat.pdf

Life sciences study design

All studies must disclose on these points even when the disclosure is negative.

Sample size	Sample size was taken as number of biological replicates
Data exclusions	No data were excluded
Replication	One replicate for RNA sequencing. Findings from RNA sequencing analysis were verified by Northern blotting. All other experiments were performed in biological triplicates unless otherwise indicated in the figure legend.
Randomization	For immunofluorescence image analysis, 10 fields of each slide were randomly selected using only the DAPI channel.
Blinding	Blinding was not relevant to our study.

Reporting for specific materials, systems and methods

We require information from authors about some types of materials, experimental systems and methods used in many studies. Here, indicate whether each material, system or method listed is relevant to your study. If you are not sure if a list item applies to your research, read the appropriate section before selecting a response.

Materials & experimental systems

n/a	Involved in the study	
☐	☒	Antibodies
☐	☒	Eukaryotic cell lines
☒	☐	Palaeontology and archaeology
☒	☐	Animals and other organisms
☒	☐	Human research participants
☒	☐	Clinical data
☒	☐	Dual use research of concern

Methods

n/a	Involved in the study	
☒	☐	ChIP-seq
☐	☒	Flow cytometry
☒	☐	MRI-based neuroimaging

Antibodies

Antibodies used

-Primary antibodies for western blot:
Actin Sigma Cat#A2066, RRID: AB_476693 Tubulin Sigma Cat#T5168, RRID: AB_477579
Total H2A.X Bethyl Cat#A300-082A, RRID: AB_203287
YH2A.X S139 Clone JBW301 Milipore Cat#05-636, RRID: AB_309864 H4 abcam Cat#ab17036, RRID: AB_1209245
U1A abcam Cat#ab166890
Lamin B1 abcam Cat#ab8982, RRID: AB_1640627

-Secondary antibodies for western blot:
IRDye 800CW Life Technologies Cat#926-32211, RRID: AB_621843 IRDye 680RD Life Technologies Cat#926-68070, RRID: AB_10956588

-Primary antibody for immunofluorescence:
YH2A.X S139 Clone JBW301 Milipore Cat#05-636, RRID: AB_309864

-Secondary antibody for immunofluorescence: Alexa Fluor 594, Life Technologies, #A11032

Validation

All antibodies were obtained commercially and had been validated by the corresponding manufacturer.

Eukaryotic cell lines

Policy information about cell lines

Cell line source(s)

-HCT116 were obtained from the Laboratory of William Marzluff (University of North Carolina at Chapel Hill, USA)

Cell line source(s)	-RPE-1 were provided by the Laboratory of Jean Cook (University of North Carolina at Chapel Hill, USA)<br>-HeLa Flp-In T-REx cells were generated and obtained from Elena Dobrikova and Matthias Gromeier (Duke University Medical Center, North Carolina, USA)<br>-Jurkat cells were obtained from the Acuto laboratory (University of Oxford, UK)<br>-iPS cell samples were provided by Sally Cowley (University of Oxford, UK)
Authentication	Only standard cell lines were employed and showed the expected growth and morphology characteristics.
Mycoplasma contamination	All cell lines tested negative for mycoplasma.
Commonly misidentified lines (See ICLAC register)	No misidentified cell lines were used in this study.

Flow Cytometry

Plots

Confirm that:

☒ The axis labels state the marker and fluorochrome used (e.g. CD4-FITC).

☒ The axis scales are clearly visible. Include numbers along axes only for bottom left plot of group (a 'group' is an analysis of identical markers).

☒ All plots are contour plots with outliers or pseudocolor plots.

☒ A numerical value for number of cells or percentage (with statistics) is provided.

Methodology

Sample preparation	-Propidium iodide staining. Cells were harvested using trypsin and thoroughly resuspended to disrupt any cell clumps. After pelleting the cells at 300 x g at room temperature, they were washed once with PBS and repelleted. The supernatant was removed and the cell pellet was resuspended in 50 µL PBS. For fixation, the tube containing the cell suspension was placed on a vortex and 1 mL 70% ethanol was added dropwise to avoid clumping. Samples were then stored at 4°C or directly processed further. Before staining, cells were washed once with PBS and then resuspended in up to 0.5 mL staining solution (PBS with 0.1% Triton X-100, 0.2 mg/mL DNAse-free RNAse A (Thermo Fisher Scientific, #10753721) and 0.02 mg/mL propidium iodide (Sigma, #P4170). After sample incubation for 30 min at room temperature in the dark, the samples were analysed by flow cytometry.<br><br>-EdU labelling. Cells were treated with 10 µM EdU (Abcam, #ab146186) for 30 min, allowing EdU incorporation into the newly synthesised DNA. As described above, cells were harvested using trypsin, washed once with PBS and thoroughly resuspended in 50 µL PBS. In this case, cells were fixed with 0.5 mL 4% formaldehyde in PBS for 15 min at room temperature. To help pellet cells after fixation, 1 mL 1% BSA in PBS was added. At this stage, samples could be stored at 4°C or directly processed further. Cells were pelleted by spinning at 1000 x g at room temperature for 3-5 min. All spins in this protocol were performed using these conditions. The supernatant was discarded and cells were resuspended in 1 mL PBS with 1% BSA and 0.5% Triton X-100 and incubated at room temperature for 15 min for permeabilisation. For staining, cells were pelleted and resuspended in 0.5 mL labeling reaction: 1 mM CuSO4 (Copper II sulfate pentahydrate, Sigma, #C8027), 0.2-2 µM Alexa Fluor 647 Azide (Triethylamoniumsalt, Thermo Fisher, #A10277), 100 mM L-ascorbic acid (Sigma, #A7506) in PBS. Importantly, the L-ascorbic acid was prepared fresh every time and only added to the labelling reaction mix immediately before use. Cells were stained for 30 min in the dark and 1 mL of PBS with 1% BSA and 0.5% Triton X-100 was added before pelleting the cells. Cell pellet was resuspended in DAPI staining solution: 1 µg/mL DAPI (Thermo Fisher Scientific, #62248), 100 µg/mL DNAse-free RNAse A (Thermo Fisher Scientific, #10753721) in PBS with 1% BSA and 0.5% Triton X-100. After incubating over night at 4°C or for 1 hr at 37°C, samples were analysed by flow cytometry.
Instrument	Samples stained with propidium iodide were analysed with the BD FACS Calibur with the 488 nm laser using a 585/42 band pass filter. Samples labelled with EdU (Alexa 647) and stained with DAPI were analysed with BD LSRFortessa X-20. DAPI was detected with the 405 nm laser, a 450/50 band pass filter and a 420 nm long pass filter. Alexa 647 was detected using a 640 nm laser, a 670/30 band pass filter and a 665 nm long pass filter.
Software	Flow cytometry data were analysed with FlowJo version 10.7
Cell population abundance	Acquisition was stopped once 10.000 single cells were counted (i.e. gated population in FSC/FSCW)
Gating strategy	First dead cells were excluded by selecting the main cell population in the FSC/SSC plot, then FSC/FSCW plots were used to gate single cells, then DAPI/cound histograms were plotted to get a cell cycle distribution graph (final plot for samples stained with propidium iodide - Supplementary Fig. 1b). For EdU labeled samples the final plot was the DAPI/AF647 where cells in G1, S and G2/M phases were quantified (Figure 2d and Supplementary Figures 1b, 5 and 7c).

☒ Tick this box to confirm that a figure exemplifying the gating strategy is provided in the Supplementary Information.
